# Changes in the provision of instrumental support by older adults in nine European countries during 2004—2015: a panel data analysis

**DOI:** 10.1186/s12877-020-01785-4

**Published:** 2020-10-31

**Authors:** S. K. Lestari, X. de Luna, M. Eriksson, G. Malmberg, N. Ng

**Affiliations:** 1 Centre for Demographic and Ageing Research, Umeå University, Umeå, Sweden; 2 Department of Epidemiology and Global Health, Umeå University, Umeå, Sweden; 3 Umeå School of Business, Economics and Statistics, Umeå University, Umeå, Sweden; 4 Department of Social Work, Umeå University, Umeå, Sweden; 5 Department of Geography, Umeå University, Umeå, Sweden; 6 Department of Public Health and Community Medicine, Institute of Medicine, University of Gothenburg, Gothenburg, Sweden

**Keywords:** Social support, Ageing, Europe, Panel data analysis, Growth model

## Abstract

**Background:**

Providing support to others has been shown to be beneficial to older adults. As people age, their health and social relationships change. These changes may also relate to changes in social support provision. We examined the trajectory of instrumental support provision by older people in three European regions throughout 11 years of follow-up. We then examined the extent to which age at baseline, sex, and region (representing welfare state regime) influenced the variations in the trajectory.

**Methods:**

Data collected from 8354 respondents who had completed at least waves 1 and 6 of the Survey of Health, Ageing and Retirement in Europe (SHARE) was analysed. Instrumental support provision was determined from asking a single question regarding whether the respondent provided help personally for people outside their household. Region, sex, and age at baseline were the main predictors tested. We used growth modelling to address the aims of this study.

**Results:**

The northern European region (Sweden and Denmark) had the highest odds ratio of instrumental support provision. The likelihood of being involved in providing instrumental support decreased by 8% annually (OR: 0.916, 95%CI: 0.893,0.940) over the 11 years of follow-up. Older respondents were less likely to provide instrumental support and their trajectories declined faster than those of the younger respondents. Sex difference in instrumental support provision was more apparent among younger-older people in the southern European region.

**Conclusions:**

Older European adults are an important source of instrumental support, especially for their families. The probability of instrumental support provision by European older adults declines over time. Age, sex, and welfare state regime predict this trajectory.

## Background

Providing personal care for others has often been associated with caregivers’ physical and mental health problems [1]. However, a substantial amount of past research attests to the positive effects of giving social support [2–5]. Giving support could increase quality of life, happiness, and feelings of belonging [3]. A longitudinal study also showed that providing support was associated with longevity [2]. On the other hand, support providers may also experience negative feelings when they are not providing the support voluntarily, or when they know that their support may not be effective in helping the support receivers [3]. Even though the effects of receiving or providing support are well-documented, studies on the determinants of support provision, especially by older adults, are limited [6]. Several factors, such as sex, health conditions, social network characteristics, and family structure may influence social support exchange [6, 7]. Furthermore, considering the declining mental and physical health, and the narrowing personal social network that older adults experience as their age advances, the likelihood of providing social support by older adults also decrease with age.

Different types of support may have different trajectories and determinants as the older adults age. A longitudinal study in the USA found that the provision of emotional support (e.g. empathy, reassurance, and trust) was stable over the 10-year follow-up while the provision of instrumental (e.g. financial assistance, help with daily tasks) and informational (e.g. advice or guidance) supports declined with age [8]. Studies that investigate changes in instrumental support provision by older adults over a long period of time in Europe are scarce [9]. In assessing instrumental support provision in the European settings, it is important to take into account the different welfare regimes and family structure across Europe. In southern European countries (e.g. Spain and Italy) where people hold the more traditional family norms and social services are less available, support exchange may be concentrated within their households. On the other hand, in countries with more generous welfare-state policies and where co-residence with adult children is less common (e.g. in Scandinavian countries such as Sweden and Denmark), it is likely that routine support exchanges include people outside their own households [10, 11]. Furthermore, the decreasing trend of co-residence of the older parents with their adult children in Europe [12] suggests that the provision of social support to other households is an important field to explore. Therefore, in this paper we used data from the Survey of Health, Ageing and Retirement in Europe (SHARE) to explore the trajectories of instrumental support provision by older European adults to people outside their own household.

SHARE is a longitudinal population study conducted biannually in most of European Union countries and Israel. SHARE focuses on health, well-being, socioeconomic, and social relationships among a European population aged 50 and over and their partners (irrespective of their age) [13]. A total of 21 European countries (including Israel) have participated in at least one wave of SHARE. The first data collection was conducted in 2004, and by 2015 six waves of data collection had been completed in nine European countries. Unlike the 3rd wave which is part of the SHARELIFE panel and assesses life history, data from the other waves belong to the regular panel [13]. Instruments used in SHARE were harmonised with other health and retirement survey such as the English Longitudinal Study of Ageing (ELSA) and the US Health and Retirement Study (HRS). Thus, not only SHARE allows comparison across countries participated in SHARE, comparison with findings from ELSA and HRS is also possible [13].

The present study used data from nine countries that participated in wave 1,2,4,5,6 of SHARE to 1) describe the trajectory of instrumental support provision by older people in three European regions throughout the 11 years of follow-up; 2) estimate the extent to which age at baseline, sex, and region influences the variations in the trajectory of instrumental support provision.

### Previous research and hypotheses

Dunkel-Schetter et al. [6] suggest that there are several conditions that promote support provision, such as when both the potential recipient and provider are in a good relationship, when the situation the potential recipient is in is appraised as being stressful, or when they accept social norms that dictate the provision of support (e.g. filial obligation). Also, social provision is more likely to occur when the support provider feels empathy toward the distressed person, feels responsible for the distressful situation, believes that the distressed person has no control over their situation, or perceives that the distressed person is actively coping with their situation or seeking for help. Willingness to provide help generally increases with the level of distress. But a high level of distress that persists over a long period of time, such as a chronic health condition, may elicit lower social support. Also, past experience of support provision is an important determinant of future support provision. Providers whose support was underappreciated, repeatedly rejected, or was not effective may be less likely to provide support again [6].

Furthermore, social support exchange is also determined by personal characteristics, sociocultural factors, socioeconomic factors, and social network characteristics [6, 7]. Social support exchange mainly occurs within the personal social network. An individual’s social network includes all their social contacts within various types of relationships, e.g. family, friends, co-workers, neighbours, and other acquaintances. Social network characteristics such as size, density, source of ties, homogeneity, and frequency of contacts, could shape the social support exchange within it [7]. Social networks that are characterised by trust, reciprocity, frequent contact, and close proximity between members promote social support provision [14]. Past study has also reported that being in larger and denser social networks enables people to provide support [15].

On the other hand, personal characteristics (e.g. age, sex, health conditions) could determine social support provision directly or indirectly through their influence on an individual’s personal social network. A cross-sectional analysis of SHARE data showed a lower prevalence of instrumental support provision in the older age group [16]. Similarly, a study in the USA reported a decline in the provision of, and an increase in the receipt of, instrumental support with age [8]. However, age itself may not have a direct effect on social support exchange [17]. Age is likely to act as a proxy for other factors such as changes in biological, physiological, or social aspects that people experience as they are getting older [17]. Therefore, to examine the changes in social support provision in ageing populations, we need to take into account other factors that, on the one hand, commonly change with age, and on the other hand, affect social support provision.

It has been suggested that older adults tend to have a smaller-sized social network. As people age, they are likely to perceive that their time is getting shorter and thus focus more on relationships that satisfy emotional regulation goals e.g. family and close confidants (socioemotional selectivity theory) [18]. This reduction in social network size may also be the result of losing peripheral network members that typically have weaker ties, due to life events e.g. job entry, marriage, parenthood, and loss of a spouse (convoy theory) [19, 20]. A decreasing social network size may indicate a reduction in the number of potential support recipients, which may lead to a lower likelihood of support provision.

As mentioned above, marital status could shape the personal social network. Social networks of married, or previously married, people have more kin-ties than the networks of unmarried people. A study among adult white Americans found that unmarried people and divorcees were more likely to exchange instrumental support with friends, neighbours, or co-workers than people who were married/had a partner or were widowed [21]. While changes in marital status mostly occur during young or mid-adult life, marital status also changes naturally in older age due to the loss of a spouse [12] and could change the characteristics of older people’s social networks and support provision.

Another key determinant of social support that is likely to deteriorate with age are physical and cognitive functions [22]. As the health of older adults is compromised, they may become more dependent and as such are more likely to receive support rather than provide it. Past studies have shown that, over time, self-rated health in North American and European countries has a declining trajectory [23]. Furthermore, prior analysis of SHARE data showed that the rate of having severe limitations in activities of daily living (ADL) gradually increases with age, with a steep increase after the age of 90 [16], indicating increasing dependency with age. Therefore, we expect that:

- Hypothesis 1: Provision of instrumental support by older adults for people (family, friends, or neighbours) outside of their household will have a declining trajectory.

Sex has a substantial influence on social networks, possibly as the result of the distinct life experiences of men and women [24]. Due to the traditional gender roles of women as homemakers and men as breadwinners, men appear to benefit more from an occupational network than women [25], while women’s social networks tend to mostly consist of friends and family [26]. Furthermore, as mothers are commonly more involved in child rearing and in their children’s education-related activities than fathers are, women may acquire more social network members from interactions with other parents [19].

The difference in social network characteristics between men and women could determine both the type of support provided and the beneficiary of it. Previous studies have reported that, compared to men, women tend to be the main care provider for the family. This pattern holds even among older adults in the more advanced age group [27, 28]. Women are more likely to be involved in emotional support and personal care than are men. On the other hand, men are more likely to exchange instrumental support (e.g. giving lifts, helping with work in the yard) than women [21, 29]. However, a previous study using SHARE data reported that across Europe, women provided more sporadic and more intensive (almost daily) instrumental support for their parents (who were not in the same household) than men did [29]. Therefore, in this study we expect that:

- Hypothesis 2: Women will have a higher likelihood of instrumental support provision for people (family, friends, or neighbours) outside of their household.

Regional or national differences, in terms of health, economic, and social aspects, have been consistently reported by studies that used SHARE data. For instance, older adults in countries characterised by a universal welfare state, e.g. northern European countries (Sweden and Denmark), reported a higher self-rated health, fewer chronic conditions, and lower physical limitations while the opposite was reported by respondents in the southern region which has the more family-based welfare models (e.g. Spain and Italy) [30, 31]. Even though the prevalence of ADL limitation increases with age in all countries, in Greece, Spain, and Italy, a considerable increase in dependency was observed between ages 50 and 70 years. A similar increase occurred among people older than 70 years in Sweden and Switzerland [32]. In terms of support provision, data from wave 1 of SHARE showed that about a third of older adults provided help for others [12]. Some regional differences were also reported, e.g. women in southern countries were more likely to provide intensive care for their dependent parents than women in other European regions [33]. Contextual factors such as culture, norms, and social policy (e.g. welfare regimes) are likely to be the driving force behind this regional difference.

Obviously, the role of family members in support and care varies across countries and regions in Europe due to the type and generosity of welfare services in different welfare regimes; i.e. the extent to which the government distributes and redistributes access to welfare and resources to the citizens, usually through its health care system and social policies such as education, social insurance, and pension programmes [34]. According to Esping-Andersen’s welfare typology [35], countries in the northern region, e.g. Sweden and Denmark, represent a ‘social democratic’ welfare state regime, i.e. a more universal and generous welfare state that is characterised by high taxes, high income redistribution, high female participation in the workforce, high standard of living, and high trust in the public system. The central region includes ‘conservative’ (i.e. Austria, Belgium, France, Germany) and ‘liberal’ welfare regimes (i.e. Switzerland). A ‘conservative’ welfare state is characterised by low female participation in the workforce, a moderate redistribution of income, higher unemployment, and dependency on social contributions. A ‘liberal’ welfare regime has a low level of total state spending, low expenditure on social services and high inequality. Italy and Spain are grouped in the southern region which has a ‘fragmented’ welfare system characterised by diverse income protection, limited or partial coverage of health services, and reliance on the family and charitable sectors [36].

Family is arguably the first line of support for its members. However, the type and the extent to which support is exchanged between family members varies considerably across countries and types of welfare regimes and is partly related to differences in values and norms. Countries in northern Europe are often grouped as “weak family countries”, while countries in southern European region are grouped as “strong family countries” [37]. This grouping is based on the strength of family loyalties, allegiances, and authority [37]. Furthermore, data from the first wave of SHARE supported this variation in family characteristics (marital status, number of living children, co-residence with adult children and frequency of contact) across Europe [11]. For instance, northern European countries (Sweden and Denmark) had the highest proportion of respondents who were divorced and, at the same time, Italy and Spain had the lowest divorce level. Co-residence with adult children was at its lowest in the northern region (around 13%) and its highest in the southern region (around 48%). This regional difference in co-residence was even higher within the oldest age group (34% in Spain vs 1% in Sweden). Similarly, daily contact with children was more common in the southern region (around 85%), followed by the central region, and the northern region (around 43%) [11]. Further analysis of SHARE data has shown that while the most common family type in Europe is familialism (characterised by a support for family obligation norm in which parents and children have frequent contact and live in close proximity), this type was more common in southern European countries. However, previous research has indicated that the strong welfare state has not necessarily resulted in a “crowding out” of family support and that instead the families have a more complementary role in northern Europe, examples of which are parents who offer support from a distance and children living apart from each other but still in frequent contact [38].

Considering that the countries with family-based models have a higher co-residence and that these countries have a relatively less welfare service, we expect that families in southern European countries will prioritise social support provision for their household members. Additionally, the more traditional family norm and the lower female participation in the workforce in southern European countries may lead to relatively larger sex differences in social support provision. On the other hand, the generous welfare services provided for people in northern European countries (“weak family countries”) may alleviate the burden of providing care for their relatives, allowing them to foster broader social networks and provide help outside their household [34]. Therefore, we hypothesised that:

- Hypothesis 3: Northern European countries will have the highest, while southern European countries will have the lowest likelihood of instrumental support provision by older adults for people (family, friends, or neighbours) outside of their household.

## Methods

### Data source

The present study used the SHARE longitudinal data (Release 6–1-0). Data from the nine countries that participated in wave 1–6 (i.e. Austria, Belgium, Denmark, France, Germany, Italy, Spain, Sweden, and Switzerland) were included in the analysis.

### Study sample

The first wave of SHARE included 29392 respondents aged ≥50 from the 12 participating countries. Among them, a total of 21483 respondent were from the nine countries. A total of 8636 respondents with a total of 40123 observations met our three inclusion criteria: 1) aged 50 and over in wave 1; 2) participated in at least waves 1 and 6; 3) have never moved to a nursing home. When respondents resided in a nursing home, they were interviewed using a different set of questionnaires, thus their data may not be comparable to data from community-dwelling respondents. Of the eligible respondents, a total of 8618 respondents (35779 observations) had at least two valid values of support provision in any two waves (a requirement for a trajectory analysis). A total of 941 observations were further excluded because of missing data in any one of the variables of interest. Thus, 34838 observations from 8354 respondents have been included in the current analysis.

### Measures

All measures used in this study were obtained by trained interviewers from face-to-face interviews using computer-assisted personal interviewing (CAPI). In addition, SHARE collected information from self-administered questionnaires and physical tests. More details on the data collection procedures is available elsewhere [13].

#### Outcome measure: instrumental support provision

In this study, instrumental support provision was defined as an act of providing any personal care tasks (e.g. dressing, bathing or showering, eating, getting in or out of bed or using the toilet), practical household help (e.g. home repairs, gardening, transportation, shopping, household chores), and/or help with paperwork (e.g. filling out forms, settling financial or legal matters) by the respondent for people from outside of their household. The provision of instrumental support was assessed from the question *“In the last twelve months, have you personally given any kind of help listed on this card to a family member from outside the household, a friend or neighbour?”*

#### Explanatory variables

The explanatory variables are *time-constant*, that is they have a constant value across the follow-up periods. *Age at baseline* was the respondent’s age in the first wave, calculated based on birth date and interview date. In the main analysis, the baseline age was rescaled, so that zero is equivalent to the lowest respondent age (50 years). *Sex* had two categories, i.e. women and men. The nine SHARE countries included in this study were grouped into three regions that represented their geographical location and welfare state regimes i.e. northern (Sweden and Denmark), central (Austria, Belgium, France, Germany, and Switzerland), and southern Europe (Spain and Italy).

#### Time measures

*Time* indicates the number of years that have passed since the baseline interview, taken as the difference between the date of first interview (wave 1) and the date of the subsequent interviews. SHARE is run biannually, but the follow-up time was not exactly the same for all countries. Thus, in this study follow-up time ranged from 0 for wave 1 to 12 for wave 6.

#### Control variables

We controlled for various sociodemographic variables and subjective health measures. Except for education level, the rest of the control variables are *time-varying variables,* the values of which were updated in each follow-up wave. *Education level* was defined as the highest level of education reported by the respondents throughout the study. We recorded education level according to the 1997 International Standard Classification of Education (ISCED-97), with seven levels ranging from 0 (early childhood education) to 6 (doctoral or equivalent level). In this study, the ISCED levels were re-categorised into “low” (ISCED 0, 1 and 2), “middle” (ISCED 3 - Upper secondary education and 4-post-secondary non-tertiary education), and “high” (ISCED 5 and 6). Employment status was ascertained from question *“In general, which of the following best describes your current employment situation?”* and the responses were grouped as “retired”, “employed” (including self-employed or working for family business), “not employed” (including unemployed, looking for work, homemaker, rentier, living off own property, student, or too sick or disabled to work). We included marital status, household size, and number of living children to take into account family structure. *Marital status* was classified as “with partner” (married or had a partner) and “no partner” (never married, divorced, or widowed). *Household size* is the total number of people residing in the respondent’s household. *Number of children* is the number of living children (including natural, fostered, adopted and stepchildren) reported by the respondent. *Self-perceived health* was measured using the question *“Would you say your health is...?”*. Respondents who reported “Excellent”, “Very good” or “Good” were classified as in “good” health, while those who reported “Fair” or “Poor” were classified as being in “poor” health.

### Statistical analyses

We used descriptive analysis to report respondents’ sociodemographic characteristics, as well as the distribution of self-perceived levels of health and provision of support. We addressed the aims of the study using a growth model with a multilevel approach. To test the fit of a multilevel growth model for our data, we first specified the unconditional models. The unconditional mean model (Model 1) was specified without any predictors to confirm whether there are ‘within-individual variations’ (changes in the odds of providing support over time for a given individual) and ‘between-individual variations’ (variation between individuals in the odds of providing support over time) [39].

Next, the unconditional means growth model (Model 2) was specified by adding the time variable. This model was used to describe the average pattern of change in the odds of instrumental support provision across the follow-up period, as well as to check the presence of the between-individual variance regarding the trajectory of instrumental support provision. In the conditional models, we added time-constant (Model 2A) and time-varying predictors (Model 2B). We also tested within level and cross-level interactions. In the final model (Model 3), we kept interaction terms that changed the time estimate.

The final model used in this study is presented in eq. 1.0 with growth parameters as specified in eq. 1.1–1.10. For a more detailed model formulation see section A in the Additional file.

1.0 $$ \log \left(\frac{\pi_{\mathbf{ij}}}{\mathbf{1}-{\pi}_{\mathbf{ij}}}\right)={\alpha}_{0\mathrm{i}}+{\alpha}_{1\mathrm{i}}{\mathrm{Time}}_{\mathrm{ij}}+{\alpha}_{2\mathrm{i}}\mathrm{No}{\mathrm{partner}}_{\mathrm{ij}}+{\alpha}_{3\mathrm{i}}{\mathrm{Householdsize}}_{\mathrm{ij}}+{\alpha}_{4\mathrm{i}}\mathrm{Numberof}{\mathrm{livingchildren}}_{\mathrm{ij}}+{\alpha}_{5\mathrm{i}}{\mathrm{Poorhealth}}_{\mathrm{ij}}+{\alpha}_{6\mathrm{i}}{\mathrm{Time}}_{\mathrm{ij}}\mathrm{x}\mathrm{No}{\mathrm{partner}}_{\mathrm{ij}}+{\alpha}_{7\mathrm{i}}{\mathrm{Time}}_{\mathrm{ij}}\mathrm{x}{\mathrm{Poorhealth}}_{\mathrm{ij}}+{\alpha}_{8\mathrm{i}}{\mathrm{Retired}}_{\mathrm{ij}}+{\alpha}_{9\mathrm{i}}{\mathrm{Notemployed}}_{\mathrm{ij}} $$

1.1 $$ {\boldsymbol{\upalpha}}_{\mathbf{0i}}={\upbeta}_{00}+{\upbeta}_{01}\mathrm{Age}\ \mathrm{at}\ {\mathrm{baseline}}_{\mathrm{i}}+{\upbeta}_{02}{\mathrm{Man}}_{\mathrm{i}}+{\upbeta}_{03}\mathrm{Middle}\ {\mathrm{education}\ \mathrm{level}}_{\mathrm{i}}+{\upbeta}_{04}\mathrm{High}\ {\mathrm{education}\ \mathrm{level}}_{\mathrm{i}}+{\upbeta}_{05}{\mathrm{Central}\ \mathrm{region}}_{\mathrm{i}}+{\upbeta}_{06}{\mathrm{Northern}\ \mathrm{region}}_{\mathrm{i}}+{\upbeta}_{07}\mathrm{Age}\ \mathrm{at}\ {\mathrm{baseline}}_{\mathrm{i}}\ \mathrm{x}\ {\mathrm{Man}}_{\mathrm{i}}+{\upbeta}_{08}\mathrm{Middle}\ {\mathrm{education}\ \mathrm{level}}_{\mathrm{i}}\ \mathrm{x}\ {\mathrm{Central}\ \mathrm{region}}_{\mathrm{i}}+{\upbeta}_{09}\mathrm{High}\ {\mathrm{education}\ \mathrm{level}}_{\mathrm{i}}\ \mathrm{x}\ {\mathrm{Central}\ \mathrm{region}}_{\mathrm{i}}+{\upbeta}_{010}\mathrm{Middle}\ {\mathrm{education}\ \mathrm{level}}_{\mathrm{i}}\ \mathrm{x}\ {\mathrm{Northern}\ \mathrm{region}}_{\mathrm{i}}+{\upbeta}_{011}\mathrm{High}\ {\mathrm{education}\ \mathrm{level}}_{\mathrm{i}}\ \mathrm{x}\ {\mathrm{Northern}\ \mathrm{region}}_{\mathrm{i}}+{\upbeta}_{012}{\mathrm{Man}}_{\mathrm{i}}\ \mathrm{x}\ {\mathrm{Central}\ \mathrm{region}}_{\mathrm{i}}+{\upbeta}_{013}{\mathrm{Man}}_{\mathrm{i}}\ \mathrm{x}\ {\mathrm{Northern}\ \mathrm{region}}_{\mathrm{i}}+{\mathrm{u}}_{0\mathrm{i}} $$

1.2 $$ {\boldsymbol{\upalpha}}_{\mathbf{1i}}={\upbeta}_{10}+{\upbeta}_{13}\mathrm{Middle}\ {\mathrm{education}\ \mathrm{level}}_{\mathrm{i}}+{\upbeta}_{14}\mathrm{High}\ {\mathrm{education}\ \mathrm{level}}_{\mathrm{i}}+{\upbeta}_{15}{\mathrm{Central}\ \mathrm{region}}_{\mathrm{i}}+{\upbeta}_{16}{\mathrm{Northern}\ \mathrm{region}}_{\mathrm{i}}+{\upbeta}_{17}\mathrm{Age}\ \mathrm{at}\ {\mathrm{baseline}}_{\mathrm{i}}\ \mathrm{x}\ {\mathrm{Man}}_{\mathrm{i}}+{\upbeta}_{18}\mathrm{Age}\ \mathrm{at}\ {\mathrm{baseline}}_{\mathrm{i}}\ \mathrm{x}\ {\mathrm{Woman}}_{\mathrm{i}}+{\mathrm{u}}_{1\mathrm{j}} $$

1.3 $$ {\boldsymbol{\upalpha}}_{\mathbf{2i}}={\upbeta}_{20}+{\upbeta}_{22}{\mathrm{Man}}_{\mathrm{i}}+{\upbeta}_{25}{\mathrm{Central}\ \mathrm{region}}_{\mathrm{i}}+{\upbeta}_{26}{\mathrm{Northern}\ \mathrm{region}}_{\mathrm{i}} $$

1.4 $$ {\boldsymbol{\upalpha}}_{\mathbf{3i}}={\upbeta}_{30} $$

1.5 $$ {\boldsymbol{\upalpha}}_{\mathbf{4i}}={\upbeta}_{40}+{\upbeta}_{45}{\mathrm{Central}\ \mathrm{region}}_{\mathrm{i}}+{\upbeta}_{46}{\mathrm{Northern}\ \mathrm{region}}_{\mathrm{i}} $$

1.6 $$ {\boldsymbol{\upalpha}}_{\mathbf{5i}}={\upbeta}_{50} $$

1.7 $$ {\boldsymbol{\upalpha}}_{\mathbf{6i}}={\upbeta}_{60}+{\upbeta}_{65}{\mathrm{Central}\ \mathrm{region}}_{\mathrm{i}}+{\upbeta}_{66}{\mathrm{Northern}\ \mathrm{region}}_{\mathrm{i}} $$

1.8 $$ {\boldsymbol{\upalpha}}_{\mathbf{7i}}={\upbeta}_{70} $$

1.9 $$ {\boldsymbol{\upalpha}}_{\mathbf{8i}}={\upbeta}_{80} $$

1.10 $$ {\boldsymbol{\upalpha}}_{\mathbf{9i}}={\upbeta}_{90} $$

Since Model 3 was specified with several interaction terms, the interpretation of those effects was not straightforward. The odds ratios do not readily communicate the nature of the interaction terms. Estimates of interaction terms need to be interpreted by considering the independent effect of each predictor in the interaction term. Thus, we calculated the predicted probability of support provision from Model 3 based on the marginal effects at representative values (MERs) method, using the *marginplots* command in Stata. We predicted the trajectory of instrumental support provision spanning over a decade for different regions and sex, and started at three baseline ages, i.e. age 50, 60, and 70.

As a sensitivity analysis, we specified Model 3 as a three-level multilevel analysis with the variable *region* in the third level. However, this model only worked without a random slope due to the limited sample size. In general, this three-level model led to the same conclusion as Model 3 which is used in the present study. We used Stata MP (Version 15.1) for all analyses.

## Results

### Respondents’ characteristics

Table 1 presents the descriptive statistics of outcome and covariate variables. Sex, education, and region are time-constant predictors, thus their different values by wave were due to the different sample sizes in each wave. In wave 1, ages ranged from 50 to 95 years old with a mean of 62.5 years. There were more female respondents in the sample (56.3%). Respondents from central Europe (Austria, Belgium, France, Germany, and Switzerland) comprised 49.6% of the sample, while the other two regions shared similar proportions. The most common highest education attainment was low level (48.0%) and the majority of respondents were retired (44.6%).

**Table 1:** Characteristics of the respondents across different waves

	Wave 1 (***n*** = 8326)	Wave 2 (***n*** = 7532)	Wave 4 (***n*** = 5194)	Wave 5 (***n*** = 5569)	Wave 6 (***n*** = 8217)
**Follow-up time in year,** ***Mean*** **±** ***SD***	0	2.3 ± 0.6	6.7 ± 0.5	8.7 ± 0.5	10.6 ± 0.5
**Age,** ***Mean ± SD***	62.5 ± 8.2	64.7 ± 8.2	69.5 ± 8.4	71.3 ± 8.3	73.0 ± 8.2
**Sex,** ***n(%)***					
Women	4691 (56.3)	4249 (56.4)	2998 (57.7)	3206 (57.6)	4641 (56.5)
Men	3635 (43.7)	3283 (43.6)	2196 (42.3)	2363 (42.4)	3576 (43.5)
**Highest education level,** ***n(%)***					
Low	3997 (48.0)	3580 (47.5)	2415 (46.5)	2588 (46.5)	3946 (48.0)
Middle	2509 (30.1)	2284 (30.3)	1594 (30.7)	1719 (30.9)	2466 (30.0)
High	1820 (21.9)	1668 (22.1)	1185 (22.8)	1262 (22.7)	1805 (22.0)
**Employment status, n(%)**					
Employed	2809 (33.7)	2147 (28.5)	924 (17.8)	732 (13.1)	727 (8.85)
Retired	3709 (44.6)	3830 (50.9)	3371 (64.9)	3962 (71.1)	6273 (76.3)
Not employed	1808 (21.7)	1555 (20.6)	899 (17.3)	875 (15.7)	1217 (14.8)
**Region,** ***n(%)***					
Southern Europe	2203 (26.5)	1943 (25.8)	1279 (24.6)	1391 (25.0)	2172 (26.4)
Central Europe	4126 (49.6)	3808 (50.6)	2693 (51.9)	2798 (50.2)	4076 (49.6)
Northern Europe	1997 (24.0)	1781 (23.6)	1222 (23.5)	1380 (24.8)	1969 (24.0)
**Marital status,** ***n(%)***					
With partner	6339 (76.1)	5627 (74.7)	3247 (62.5)	3315 (59.5)	5526 (67.3)
No partner	1987 (23.9)	1905 (25.3)	1947 (37.5)	2254 (40.5)	2691 (32.8)
**Self-perceived health,** ***n(%)***					
Poor	1916 (23.0)	2217 (29.4)	1792 (34.5)	1958 (35.2)	3070 (37.4)
Good	6410 (77.0)	5315 (70.6)	3402 (65.5)	3611 (64.8)	5147 (62.6)
**Household size,** ***Mean ± SD***	2.3 ± 1.0	2.3 ± 1.0	1.9 ± 0.8	1.9 ± 0.8	1.9 ± 0.8
**Number of children,** ***Mean ± SD***	2.2 ± 1.4	2.3 ± 1.4	2.2 ± 1.4	2.2 ± 1.4	2.2 ± 1.4
**Provided social support,** ***n(%)***	3185 (38.3)	2872 (38.1)	1584 (30.5)	1633 (29.3)	2247 (27.4)

Across waves, the mean of the household size decreased while the number of living children was quite constant. Throughout the follow-up period, more respondents reported being “with partner”. However, an increasing trend of “no partner” (23.9% in wave 1 to 32.8% in wave 6) was observed. Most respondents (77.0%) perceived their health as ‘good’ in wave one and, as time passed, more respondents reported ‘poor’ health (37.4% in wave 6). As for the main outcome of this study, at baseline, 38.3% respondents reported that they provided support for persons outside their household. This number declined constantly to 27.4% in wave 6.

Additionally, data on the relationship between respondent and support recipient was available in all the waves analysed here. But data on the type of support was only available in waves 1, 2 and 6 (see Table B1 and B2 in the Additional file). The most common type of support reported was practical household help, followed by paperwork-related help and personal care. Provision of personal care and paperwork-related help was mostly reported by respondents in the southern region, while providing practical household help was more common in the northern region. In all regions the main recipients of support were respondents’ parents, children, and current or ex-partners.

### Model fit for instrumental support provision trajectory

Table 2 presents the estimates obtained from three multilevel growth models. The intercept in the unconditional mean model (Model 1) showed that the overall odds of providing instrumental support (across time and individuals) was 0.389 (*p* = 0.000). Furthermore, the variance of intercept indicated a variation between individuals as to the likelihood of instrumental support provision by older European adults over time. Thus, the fit of the multilevel growth model for our data was established. The intraclass correlation (ICC) indicated that around 34% of the total variance in the odds of instrumental support provision was due to the between-individual variation.

**Table 2:** Result of unconditional mean model (Model 1), unconditional growth model (Model 2) and final model (Model 3)

	Model 1: Unconditional mean model (***n***^**b**^ = 34838)		Model 2: Unconditional growth model (***n***^**b**^ = 34838)		Model 3 (n^**b**^ = 34838)	
	Odds Ratio (95% Confidence Interval)		Odds Ratio (95% Confidence Interval)		Odds Ratio (95% Confidence Interval)	
***Fixed effects***						
**Part i: For intercept**						
**Intercept**	0.389***	(0.374,0.405)	0.526***	(0.494,0.559)	1.283	(0.993,1.659)
**Sex**						
Women					1	
Men					0.881	(0.747,1.039)
**Baseline age**					0.930***	(0.921,0.940)
**Sex x Baseline age**						
Women x baseline age					1	
Men x baseline age					1.017**	(1.005,1.028)
**Education**						
Low					1	
Middle					1.099	(0.938,1.288)
High					1.271**	(1.067,1.513)
**Employment status**						
Employed					1	
Retired					1.301***	(1.173,1.443)
Not employed					1.109	(0.982,1.252)
**Region**						
Southern Europe					0.597***	(0.454,0.784)
Central Europe					1	
Northern Europe					1.373*	(1.039,1.813)
**Marital status**						
With partner					1	
No partner					0.992	(0.822,1.197)
**Number of living children**					0.978	(0.940,1.018)
**Self-perceived health**						
Poor					1	
Good					1.122	(0.992,1.269)
**Household size**					0.916***	(0.872,0.963)
**Sex x Marital status** ^**a**^						
Women x with partner					1	
Men x no partner					0.741**	(0.619,0.888)
**Region x Sex** ^**a**^						
Central x women					1	
South x men					0.692***	(0.561,0.853)
North x men					1.228*	(1.017,1.484)
**Region x Education** ^**a**^						
Central x low					1	
South x middle					1.640***	(1.260,2.136)
South x high					1.269	(0.897,1.795)
North x middle					1.106	(0.885,1.382)
North x high					0.95	(0.752,1.199)
**Region x Marital status** ^**a**^						
Central x with partner					1	
South x no partner					0.631*	(0.437,0.911)
North x no partner					1.547**	(1.144,2.091)
**Region x Number of children** ^**a**^						
South x number of children					0.917*	(0.853,0.987)
Central x number of children					1	
North x number of children					1.041	(0.973,1.114)
**Part ii: For time slope**						
**Time**			0.926***	(0.917,0.934)	0.916***	(0.893,0.940)
**Sex x Age x Time**						
Women x age x time					0.998**	(0.997,1.000)
Men x age x time					0.998***	(0.997,0.999)
**Region x Time**						
South x time					0.975*	(0.952,0.998)
Central x time					1	
North x time					0.991	(0.970,1.012)
**Education x Time**						
Low x time					1	
Middle x time					1.011	(0.993,1.030)
High x time					1.021*	(1.001,1.042)
**Self-perceived health x Time**						
Poor x time					1	
Good x time					1.030***	(1.013,1.048)
**Marital status x Time**						
With partner x time					1	
No partner x time					1.026*	(1.003,1.050)
**Marital status x Region x Time** ^**a**^						
With partner x central x time					1	
No partner x south x time					1.035	(0.987,1.085)
No partner x north x time					0.952*	(0.916,0.990)
***Random Effect***						
Intercept	1.719	(1.592,1.857)	2.873	(2.517,3.279)	2.26	(1.954,2.613)
Time slope			0.023	(0.019,0.028)	0.021	(0.017,0.026)
Covariance			−0.119	(−0.154,-0.083)	− 0132	(−0.166,-0.099)
***Intraclass correlation***	0.343		0.466		0.407	

* *p* value < 0.05, ** *p* value < 0.01, ***p < 0.001

^a^ The estimates for interaction that include a reference category were not shown as it equal to the main effect of the other variable. For example, estimates for interaction between central x middle education level and central x high education level are equal to estimates for middle education level (OR:1.099, 95%CI:0.938,1.288) and high education level (OR: 1.271, 95%CI:1.067,1.513), respectively

^b^ n = number of records

In the unconditional growth model (Model 2), time was added as both fixed effect and random slope effect, i.e. the effect of time could vary by individual. This model showed that the odds of providing instrumental support declined by approximately 7% on an annual basis (OR: 0.926, 95% CI: 0.917–0.934). The intercept and time variance indicated a variability in the initial likelihood and trajectory of instrumental support provision. We proceeded further by including explanatory and control variables in our model to explain these variations.

Compared to Model 2, variances for intercept and slope in Model 3 were slightly lower. This means that the addition of predictors in this model could explain only a small part of variance observed in the unconditional growth model. The negative covariance indicated that people with a high initial likelihood of instrumental support provision had a steeper decline in their trajectory. Furthermore, the ICC also reduced from 0.466 (Model 2) to 0.407 (Model 3).

### Predictors of instrumental support provision at baseline

The first part of Model 3’s estimates in Table 2 showed factors associated with instrumental support provision at baseline. We used an interaction plot to visualise the effect of covariate interactions while holding other covariates at their reference value (section C in the Additional file). Compared to the central region, the odds of instrumental support provision were 40% higher in the northern region (OR: 1.373, 95%CI:1.039,1.813) and 40% lower in the southern region 0.597 (0.454,0.784) (Table 2). The association between sex and provision of instrumental support was varies by region and age at baseline. In the southern and central region, women had higher odds than men, while in the northern region the opposite was observed (Fig. C1 in the Additional file). Furthermore, among respondents age 50 at baseline, women were more likely to provide instrumental support. On the other hand, the reverse was true among respondents age 70 at baseline (Fig. C2 in the Additional file).

The effect of marital status differed by sex and region (Table 2). In the central region, both marital statuses, “with partner” and “no partner”, had the same odds of instrumental support provision. But in the northern region, people without a partner had higher odds of instrumental support provision, while the opposite was observed in the southern region (Fig. C3 in the Additional file). Among women, marital status did not affect the provision of instrumental support. However, men who did not have partner had lower odds of instrumental support provision (Fig. C4 in the Additional file).

The effects of household size in all regions were the same, i.e. a unit increase in the number of household members was associated with an approximate 8% decrease in the odds of instrumental support provision for people outside the household (Table 2). In all regions, people with middle or high education level were more likely to provide support for people outside their household (Fig. C5 in the Additional file). As for employment status, compared to people who were employed, retirees had 1.3 times higher odds of providing instrumental support. Being not employed did not significantly increase the odds of instrumental support provision. Our results also showed that the association between self-reported health with the odds of providing support at baseline was not significant (Table 2).

### The trajectory of instrumental support provision

The second part of the final model’s results showed that the odds of instrumental support provision decrease by around 8% annually (OR: 0.92, 95%CI:0.89;0.94). The predictors of this trajectory were tested through its interaction with the time variable (Part ii of Table 2). In order to further examine the trajectories of instrumental support, we focused on our main explanatory variables, i.e. sex, age at baseline and region.

**Fig. 1:**
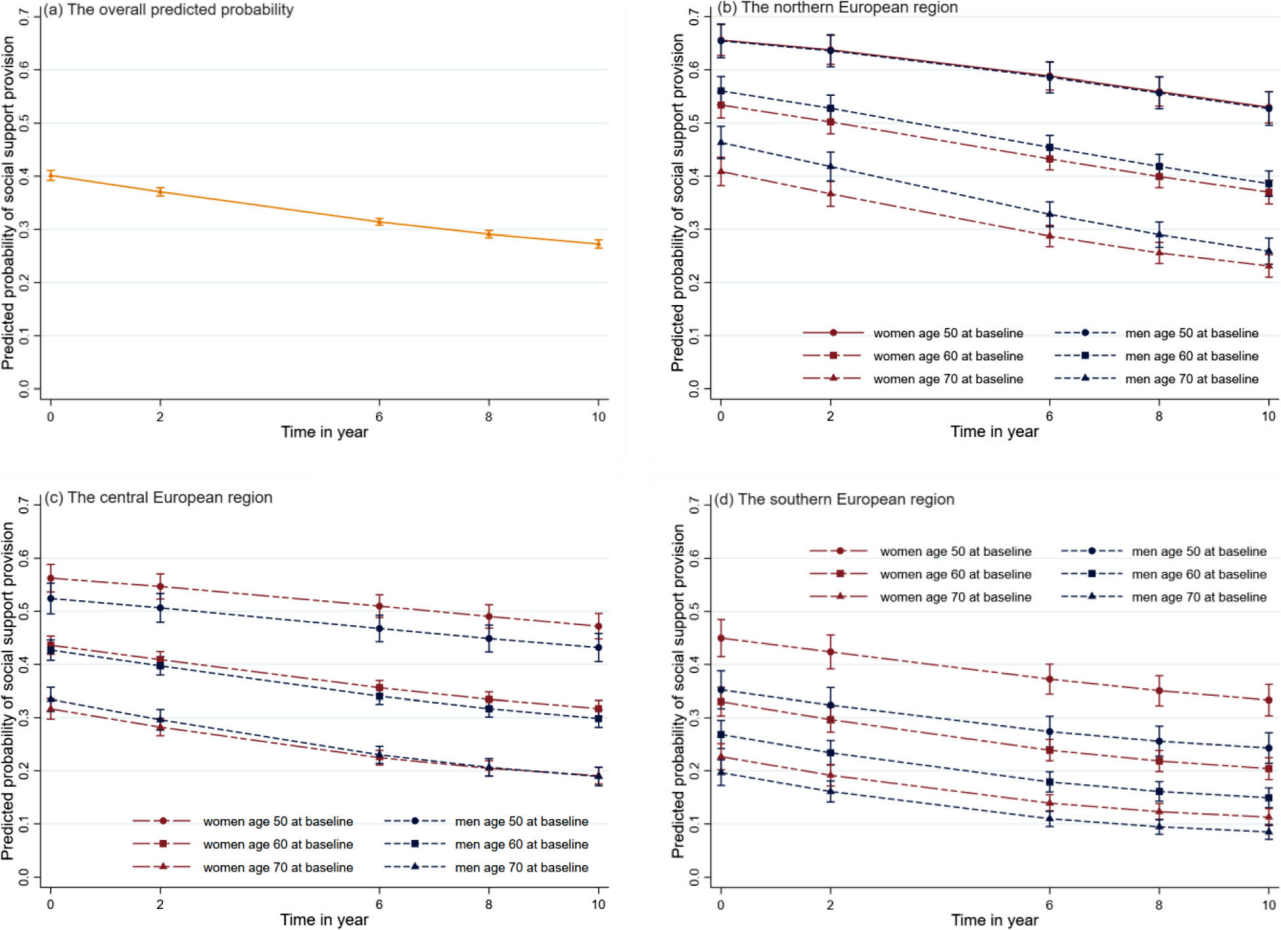
The predicted probability of instrumental support provision, (**a**) in the overall population and by sex at baseline age 50, 60, and 70 years in northern (**b**), central (**c**) and southern (**d**) European region, while the rest of predictors were held at their observed value

Figure 1 presents the trajectories of predicted probability of instrumental support provision for other households. At baseline, the overall probability was 0.40 (95%CI 0.39;0.40). In the course of 10 years, the probability declined steadily to 0.27 (95%CI 0.26;0.28) (Fig. 1(A)). The trajectories for each region by sex and three baseline ages are shown in Fig. 1(b)-(d). Generally, older people in the northern region had a higher probability of providing support than their counterparts in the central and southern regions. One common characteristic across regions was that younger baseline ages had a higher probability of providing support. In each region, women aged 50 had the highest probability of instrumental support provision at the baseline, with a probability of 65.5% in the northern, 56.2% in the central, and 45.0% in the southern region.

In the northern European region, among people aged 50 years at baseline, women and men had a very similar probability of providing support, but among those with baseline ages of 60 and 70 years old, men had a higher probability of providing instrumental support than women (Fig. 1(b)). We observed a different pattern of sex difference in the levels and trajectories in the central region (Fig. 1(c)). Women in the central European region were more likely to provide instrumental support. However, among the older baseline age cohort (age 70) men had a slightly higher probability of providing instrumental support. The northern and central region showed an opposite pattern of probability of instrumental support provision in that sex difference was larger in older baseline age cohort among Northern Europeans, while the opposite was observed among Central Europeans. In the northern and central regions, the trajectory of probability of instrumental support provision differed by baseline age, with older age being associated with a steeper decline. For the older age group (70 years old at baseline), the probability of men providing support declined faster than it did for women.

Among people in the southern region, women had a higher probability of instrumental support provision compared to men in all three of the observed baseline age cohorts. Sex difference was more apparent among Southern Europeans aged 50 and 60 at baseline. The greater the age at baseline, the smaller the difference between men and women (Fig. 1(d)). Unlike in the other regions, the rate of decline in instrumental support provision was very similar across different sexes and baseline ages.

## Discussion

Past studies have reported that older adults in European countries were important providers of support for their families. Due to changes in family structure, e.g. decreasing trend of co-residence with adult children, this support exchange is likely to involve other households. The present study explored the trajectories of European older adults’ instrumental support provision for people outside of their household. Data was obtained from five waves of SHARE data spanning a period of 11 years in nine European countries.

In this study, we tested three hypotheses. Our first hypothesis, that provision of instrumental support by older adults for people outside their household had a declining trajectory, was supported by our findings. Furthermore, we showed that the rate of this decline was gradual, and this trajectory was associated with sex, age at baseline, education level, marital status, self-perceived health, and region. As for the second hypothesis, that women had a higher likelihood of providing instrumental support for people outside of their household, was only partially supported as the association between sex and provision of instrumental support differed by age and region. On the other hand, our findings supported the third hypothesis that northern European countries had the highest, while southern European countries had the lowest likelihood (predicted probability) of instrumental support provision for people outside their household.

In the present study, we further examining the trajectory of instrumental support provision for people outside of respondents’ households by three baseline ages to tease out the possible cohort effect from ageing effect that was represented by follow-up time [40]. Our results show that, regardless of baseline age, the trajectories of instrumental support provision were declining. The initial levels, however, differed by baseline age cohort and region. In all regions, the younger cohort had the higher probability of providing instrumental support compared to the older cohorts. These differences are likely to be related to the life phase they were in during the follow-up period. Between the ages of 50–60, most of the respondents were still likely to be working [41], in good health [42], married [12], and with their parents still living. Thus, they may experience a higher ‘demand’ of support from their older generations (e.g. parents) and younger generations (e.g. children and grandchildren) [28, 43]. This demand, supported by relatively good health and socioeconomic status, results in the higher likelihood of support provision. In line with our findings, studies in the United Kingdom population reported that support provision was at its highest between ages 45–59 [43]. Similarly, ages 50–65 have been reported as comprising the peak period of family support provision in the European population [16].

As for the second age cohort, ages 60–70, people were likely to still have a spouse or partner [12], those who were employed were transitioning to retirement [41] with the potential for more leisure time to provide support for others. As shown by our findings that being retired was associated with higher odds of providing instrumental social support. Thus, people in this age group were still likely to be active providers of support. The lower instrumental support provision we observed was likely related to morbidity and physical limitations that are expected to be higher during the follow-up period of this cohort compared to the younger cohort [42].

For the older cohort, 70–80 years old, the prevalence of more severe morbidity, as well as ADL limitations, are expected to increase [42]. On the other hand, the number of their potential support recipients was likely to decrease due to the loss of their spouse and/or parents [12]. Therefore, it was expected that we would observe lower probability of instrumental support provision in this cohort. The more rapid trajectory of this cohort was also likely to be related to poor health. As observed in this study, poor self-perceived health was associated with a faster decline in the probability of instrumental support provision over time.

Turning to regional differences, the northern region had, as we predicted, the highest probability of instrumental support provision, followed by the central and southern regions. The higher average incomes in the northern region countries, as compared to those in the southern region, may increase the possibilities for providing instrumental support. Moreover, the more generous welfare services in the central and northern regions may have lessened the ‘obligation’ to provide support for family, thereby making any provision of support for people outside their household more likely. It might be expected that more instrumental support for non-household members in countries with strong welfare systems would be directed to neighbours and friends. Interestingly, in all regions, close family (children, parents, and spouses) remained as the main beneficiaries of support throughout the 11 years of follow-up of this study. These findings also indicated that family solidarity was still strong in countries with generous welfare states. In these countries, welfare services complement rather than crowd-out family solidarity, which is in line with previous research in Europe [38, 44, 45]. This arrangement led to shared responsibility between family and state [46] and resulted in a specialisation of the type of intergenerational support exchanged [45, 47]. Using SHARE data, Schmid et al. reported that in countries with more generous welfare services, adult children, regardless of their sex, were more likely to provide sporadic support [29], while the more intensive support could be provided by the government.

Furthermore, we found that the association between the number of children the respondents had and the odds of them providing instrumental support for people from other households was negative in the southern region, but positive in the northern and central regions. Considering these findings and taking into account the higher co-residence (with parents or adult children) in the southern region, it seems that the lower prevalence of support provision for people from other households observed in the southern region was due to the likelihood of their main support-beneficiaries (children, parents and spouse) living with them in the same household.

Regional differences were also found in the association between sex and instrumental support provision. Only in the southern region, women were more likely than men to provide instrumental support in all three baseline age cohorts, providing partial support for our hypothesis. Sex difference, especially among the younger age cohort, was more apparent in the southern region. In the other two regions, men had a higher probability of providing support than women among those with a baseline age of 60 and 70 years in the northern region and 70 years in the central region. This is likely to be an effect of ageing rather than cohort, as another study among the older population in the UK reported that after the age of 70, men had a higher prevalence of support provision than women [43]. These findings indicated that older women in all regions provided more instrumental support to people outside their household during the peak of the support-giving period, while older men (except those in the southern region) were likely to provide more instrumental support in the later age.

The more family-based model for care and support together with the more limited welfare service seen in the southern region [48] are likely to be associated with the higher provision of instrumental support by women. Different welfare policies implemented in the northern and central regions may contribute to the different patterns observed. Welfare policies have been shown to preserve or reduce sex inequality in support provision [29]. Sex difference in support provision may also be related to the type of support provided, e.g. women more likely than men to provide personal care. The present study did not look into specific types of instrumental support. However, our additional analysis showed that the southern region had the highest prevalence of personal care provision. This may contribute to the sex difference in instrumental support provision we observed in this region.

Finally, our study showed quite a large inter-individual variation in the level and trajectory of instrumental support provision. This inter-individual variation remains in the sensitivity analysis that used ‘region’ as the third level in the multilevel analysis. These results indicate that while country-specific conditions and welfare state policies may influence personal relationships, instrumental support exchange is a personal experience that is highly affected by personal factors and circumstances.

### Strengths and limitations

The main strength of the present study is the use of five waves of panel data from SHARE. Panel study records all changes at individual level throughout the follow-up time, therefore the shift in the results may reflect the real change in the phenomena studied. In our case, the trajectory of instrumental support provision observed may reflect the real decrease in instrumental support provision as people age, rather than the difference in sample characteristics. In addition, all countries that participated in SHARE used a standardised questionnaire, allowing for a valid comparison across countries.

However, we also acknowledge that all variables in this study are self-reported, thus true values may be under- or over-estimated. The value of self-perceived health, which has been widely used, has also been scrutinised for its sensitivity to bias. People with different cultures and languages may have different perspectives in rating their health. For example, compared to Danes and Swedes, Germans need to be much healthier to rate their health as very good [49]. However, we argue that the use of self-perceived health in the present study did not reduce the validity of our results, as our study does not aim to compare health status across European regions. Additionally, even though we found that the prevalence of good self-perceived health varied across regions, it had the same effect in all regions. The present study showed that self-perceived health did not affect the initial level but affected the trajectories of instrumental support provision. Future study could attempt to explain the initial level of social support provision by taking into account the objective measure of health status and physical function of older adults.

Instrumental support provision in this study was measured using a single-item measure. Therefore, our results need to be interpreted with caution as we may not capture all dimensions of instrumental support provision that could be measured using more comprehensive instruments. Also, the present study was unable to include social networks-related variables as such variables were only available in waves 4 and 6 of SHARE. Thus, future studies on social support exchange among older adults may use the more comprehensive measure of social support. Adding social networks characteristics such as social network size and type of relationships could improve the understanding about the determinants of changes in social support provision by older adults. Furthermore, by taking into account the different types of instrumental support in the analysis, future studies may be able to explain the sex difference in instrumental support provision. Further examination on societal level factors is also needed to explain the regional differences indicated in the present study.

Another limitation of this study was related to attrition that could lead to selection bias. Thus, limiting the generalisability of the presented results. Around 3.3% of the eligible sample (age 50 or over in wave 1, participated in at least wave 1 and 6, and have never moved to a nursing home) in the present study had missing data in the variables studied and were excluded from analysis. The final study sample and the excluded eligible sample (samples with missing data in any of variables of interest) shared a similar baseline characteristic in terms of age, sex, education, self-perceived health, number of children, and the level of instrumental support provision. The final sample, however, had a slightly lower mean household size (2.5 vs. 2.3) and a lower proportion of people with a partner (87.5% vs. 76.2%) (Table E1, Section E in the Additional file). Compared to the initial SHARE sample (age ≥ 50 in the nine countries), the final sample had a higher proportion of female respondents, was relatively younger, had higher education, and had a higher prevalence of good self-perceived health (Table E2, Section E in the Additional file).

## Conclusion

Around a third of European older adults across the continent were actively providing instrumental support for people (especially family) outside their household. The probability of instrumental support provision by European older adults had a gradual decline in its trajectory over its 11-year follow-up. The levels and trajectories of instrumental support provision differed by region, sex, and age at baseline. The probability of providing instrumental support was the highest in the northern European region and lowest in southern European region. Clear sex difference was only observed among the younger-older Southern Europeans in that women had higher probably of providing instrumental support. Past studies have shown the possible beneficial effect of providing support for older people's health and well-being. Thus, it has been suggested that interventions that encourage older people to help others were needed. Our findings indicated that such intervention may need to be tailored differently for different age groups, sex, and region. On the other hand, considering the negative effects of providing support (especially personal care) that were also well documented; and the higher level personal care type of support reported by Southern European older adults in the present study, further studies on the benefit of providing instrumental support need to take into account the different type of instrumental support as well as regional and sex differences.

## Supplementary information

**Additional file 1.** Additional analyses of instrumental support trajectories in EU. Additional information in this file includes: A. Growth model formulation. B. Type of instrumental support and recipients of instrumental support. C. Interaction Plots. D. Predicted probability plots. E. Characteristics of sample and non-sample.

## Data Availability

SHARE data is distributed by SHARE-ERIC (Survey of Health, Ageing and Retirement in Europe – European Research Infrastructure Consortium). It is freely available for scientific community for scientific research upon registration. The data usage is subject to European Union and national data protection laws as well as the SHARE *Conditions of Use*. See www.share-project.org/data-access for further information on data access.

## Abbreviations

CAPI: Computer-Assisted Personal Interviewing
CI: Confidence interval
ICC: Intraclass Correlation
ISCED: International Standard Classification of Education
MER: Marginal Effects at Representative Values
OR: Odds Ratio
SHARE: Survey of Health, Ageing and Retirement in Europe
