# Supplementary material for: Changes in the provision of instrumental support by older adults in nine European countries during 2004—2015: a panel data analysis
Source: BMC Geriatr. 2020 Oct 31;20:436. doi: 10.1186/s12877-020-01785-4 (PMC7603660; doi:10.1186/s12877-020-01785-4)
Supplement: Supplementary file 1 — Additional file 1. Additional analyses of instrumental support trajectories in EU. Additional information in this file includes: A. Growth model formulation. B. Type of instrumental support and recipients of instrumental support. C. Interaction Plots. D. Predicted probability plots. E. Characteristics of sample and non-sample. [file 12877_2020_1785_MOESM1_ESM.pdf]

Additional file for "*Changes in the Provision of Instrumental Support by Older Adults in Nine European Countries during 2004—2015: A panel data analysis*"

Lestari SK[1,2], de Luna X[3], Eriksson M[4], Malmberg G[1,5], Ng N[2,6]

[1] Centre for Demographic and Ageing Research, Umeå University, Umeå, Sweden
[2] Department of Epidemiology and Global Health, Umeå University, Umeå, Sweden
[3] Umeå School of Business, Economics and Statistics, Umeå University, Umeå, Sweden
[4] Department of Social Work, Umeå University, Umeå, Sweden
[5] Department of Geography, Umeå University, Umeå, Sweden
[6] Department of Public Health and Community Medicine, Institute of Medicine, University of Gothenburg, Gothenburg, Sweden
Corresponding author: Septi kurnia lestari (Email: s.kurnia.lestari@gmail.com)

A. Model Formulation

Multilevel approach on growth model

The basic growth model for assessing changes in the outcome across time can be decomposed into two levels:

1. Level 1 (within person)

This model has two individual growth parameters, α_{0i} and $\alpha_{1i.}$ The first growth parameter (α_{0i}) represents a given person's initial log-odds of the outcome at baseline (Time=0). The second growth parameter (α_{1i}) represents the rate of change in the log-odds of the outcome across time for that person.

$$\log\left(\frac{\pi_{ij}}{1-\pi_{ij}}\right) = \alpha_{0i}+\alpha_{1i}\text{Time}_{ij} \qquad \text{(a1.1)}$$

Where "π_{ij}" is the probability of answer positively to the social support question for individual *i* at measurement occasion *j*.

2. Level 2 (Between person)

$$\alpha_{0i}= \beta_{00}+\beta_{01}X_i+u_{0j} \qquad \text{(a2.1)}$$

$$\alpha_{1i}= \beta_{10}+\beta_{11}X_i+u_{1j} \qquad \text{(a2.2)}$$

The level 1 growth parameters are the outcomes of level 2 models. Equation a2.1 expresses the association between the first growth parameter and the time-constant predictor (X_i). While equation a2.2 determines whether the second growth parameter varies by the value of time-constant predictor across time. Both level-2 models have fixed effects and random effects. The fixed effects in equation a2.1 represents the average initial odds of providing support when predictors equal to zero (β_{00}) and the effect of predictor on the initial odds of the outcome (β_{01}).

In equation a2.2 β_{10} express the average rate of change in the odds of support provision when the predictor (X_i) equal to its reference value. While β_{11} capture the, on average, relationship between the

growth rate with the predictor. The random part in equation a2.1, random intercept (u_{0j}), represent the individual deviation from β_{00}. The random slope (u_{1j}) represent individual deviation from the population trajectory.

Specifying Multilevel Growth Model for the present study

To test the fit of multilevel growth model for our data, we first specified the unconditional models then added time-varying and time-constant predictors. We also tested within level and cross level interactions.

Unconditional mean model

The unconditional mean model was specified without any predictors to confirm whether there are 'within-individual variations' (changes in the odds of providing support over time for a given individual) and 'between-individual variations' (variation between individuals in the odds of providing support over time)

$$\log\left(\frac{\pi_{ij}}{1-\pi_{ij}}\right) = \alpha_{0i} \quad \text{(a3.1)}$$

Note:

$\log\left(\frac{\pi_{ij}}{1-\pi_{ij}}\right)$ is the log-odds that y_{ij} equals to one (positive answer to the Providing support question)

$\pi_{ij}=E(y_{ij}|x_{ij},u_j)$

$$\alpha_{0i}= \beta_{00}+u_{0j} \quad \text{(a3.2)}$$

Unconditional means growth model

This model included only the outcome and time as predictor to establish whether Time variable is associated with the odds of providing support. Equation a1.1 shows the level 1 unconditional means growth model. Models for growth parameters in this model are shown in equations a3.2 and a3.3.

$$\alpha_{1i}= \beta_{10}+u_{1j} \quad \text{(a3.3)}$$

Adding time-constant predictors

At this stage, we added time-constant covariates to the unconditional growth model by substituting the growth parameters in equation a1.1 with equations a4.1 and a4.2

$$\alpha_{0i}= \beta_{00}+\beta_{01}\text{Age at baseline}_i+\beta_{02}\text{Man}_i+\beta_{03}\text{Middle edu. level}_i+\beta_{04}\text{High edu. level}_i+ \beta_{05}\text{Central region}_i+ \beta_{06}\text{Northern region}_i+u_{0i} \quad \text{(a4.1)}$$

$$\alpha_{1i}= \beta_{10}+\beta_{11}\text{Age at baseline}_i+\beta_{12}\text{Man}_i+\beta_{13}\text{Middle edu. level}_i+\beta_{14}\text{High edu. level}_i+ \beta_{15}\text{Central region}_i+ \beta_{16}\text{Northern region}_i+u_{1j} \quad \text{(a4.2)}$$

Adding time-varying predictors

The time-varying covariates are added in equation a1.1, as in

$$\log\left(\frac{\pi_{ij}}{1\text{-}\pi_{ij}}\right) = \alpha_{0i}+\alpha_{1i}\text{Time}_{ij}+\alpha_{2i}\text{No partner}_{ij}+\alpha_{3i}\text{Household size}_{ij}+ \alpha_{4i}.\text{ Number of living children}_{ij}+\alpha_{5i}\text{Poor health}_{ij}+\alpha_{6i}\text{Time}_{ij}\text{ x No partner}_{ij}+\alpha_{7i}\text{Time}_{ij}\text{ x Poor health}_{ij}+ \alpha_{8i}\text{Retired}_{ij}+\alpha_{9i}\text{Not employed}_{ij} \qquad \text{(a5.0)}$$

With the growth parameters α_{0i} and α_{1i} as in Equation a4.1 and a4.2, while the parameters for other time-varying predictors are only fitted as fixed effect.

Interaction between predictors

We tested interaction between age at baseline with the other time-constant predictors as well as between region and other predictors. We keep interactions terms which were significant or changed the time estimate. For the final model, in the level 1, we specified interaction between time with marital status and time with self-perceived health. While the level 2 model include interactions between age at baseline with sex, region with sex and highest education level. The final model (Model 5) used in the current study is as follows:

$$\log\left(\frac{\pi_{ij}}{1-\pi_{ij}}\right) = \beta_{00} + \beta_{01}\text{Age at baseline}_i + \beta_{02}\text{Man}_i + \beta_{03}\text{Middle edu. level}_i + \beta_{04}\text{High edu. level}_i + \beta_{05}\text{Central region}_i + \beta_{06}\text{Northern region}_i + \beta_{20}\text{ No partner}_{ij} + \beta_{40}\text{N. living children}_{ij} + \beta_{50}\text{ Poor health}_{ij} + \beta_{30}\text{ Household size}_{ij} + \beta_{22}\text{Man}_i\text{xNo partner}_{ij} + \beta_{07}\text{Age at baseline}_i\text{ x Man}_i + \beta_{08}\text{Middle edu. level}_i\text{ x Central region}_i + \beta_{09}\text{High edu. level}_i\text{ x Central region}_i + \beta_{010}\text{Middle edu. level}_i\text{ x Northern region}_i + \beta_{011}\text{High edu. level}_i\text{ x Northern region}_i + \beta_{012}\text{Man}_i\text{ x Central region}_i + \beta_{013}\text{Man}_i\text{ x Northern region}_i + \beta_{25}\text{Central region}_i\text{x No partner}_{ij} + \beta_{26}\text{Northern region}_i\text{ x No partner}_{ij} + \beta_{45}\text{Central region}_i\text{ xN. living children}_{ij} + \beta_{46}\text{Northern region}_i\text{ xN. living children}_{ij} + \beta_{10}\text{Time}_{ij} + \beta_{17}\text{Age at baseline}_i\text{ x Man}_i\text{x Time}_{ij} + \beta_{18}\text{Age at baseline}_i\text{ x Woman}_i\text{x Time}_{ij} + \beta_{15}\text{Central region}_i\text{x Time}_{ij} + \beta_{16}\text{Northen region}_i\text{x Time}_{ij} + \beta_{13}\text{Middle edu. level}_i\text{x Time}_{ij} + \beta_{14}\text{High edu. level}_i\text{x Time}_{ij} + \beta_{70}\text{Time}_{ij}\text{ x Poor health}_{ij} + \beta_{60}\text{ Time}_{ij}\text{ x No partner}_{ij} + \beta_{65}\text{Central region}_i\text{x Time}_{ij}\text{ x No partner}_{ij} + \beta_{66}\text{Northern region}_i\text{ x Time}_{ij}\text{ x No partner}_{ij} + \beta_{80}\text{ Retired}_{ij} + \beta_{90}\text{ Not employed}_{ij} + u_{0i} + u_{1j}\text{Time}_{ij} \qquad \text{(a6.0)}$$

Table A1. Model Building

	**Model 1: Unconditional mean model (n=34838)** Odds Ratio (95% Confidence Interval)		**Model 2: Unconditional growth model (n=34838)** Odds Ratio (95% Confidence Interval)		**Model 2A: + time-constant variables (n=34838)** Odds Ratio (95% Confidence Interval)		**Model 2B: + time variant variables (n=34838)** Odds Ratio (95% Confidence Interval)		**Model 3 (n=34838)** Odds Ratio (95% Confidence Interval)	
Fixed effects										
**Part i: For intercept**										
**Intercept**	0.389***	(0.374,0.405)	0.526***	(0.494,0.559)	0.965	(0.828,1.124)	1.005	(0.804,1.255)	1.283	(0.993,1.659)
**Sex**										
Women					1	(1.000,1.000)	1	(1.000,1.000)	1	(1.000,1.000)
Men					0.979	(0.876,1.095)	0.973	(0.868,1.090)	0.881	(0.747,1.039)
**Baseline age**					0.948***	(0.942,0.955)	0.936***	(0.928,0.944)	0.930***	(0.921,0.940)
**Sex x Baseline age**										
Women x baseline age									1	(1.000,1.000)
Men x baseline age									1.017**	(1.005,1.028)
**Education**										
Low					1	(1.000,1.000)	1	(1.000,1.000)	1	(1.000,1.000)
Middle					1.259***	(1.098,1.444)	1.234**	(1.076,1.415)	1.099	(0.938,1.288)
High					1.329***	(1.143,1.545)	1.306***	(1.123,1.519)	1.271**	(1.067,1.513)
**Employment status**										
Employed							1	(1.000,1.000)	1	(1.000,1.000)
Retired							1.320***	(1.190,1.464)	1.301***	(1.173,1.443)
Not employed							1.131*	(1.002,1.276)	1.109	(0.982,1.252)
**Region**										
South					0.416***	(0.357,0.483)	0.457***	(0.391,0.533)	0.597***	(0.454,0.784)
Central					1	(1.000,1.000)	1	(1.000,1.000)	1	(1.000,1.000)
North					1.877***	(1.640,2.148)	1.865***	(1.628,2.137)	1.373*	(1.039,1.813)
**Marital status**										
With partner							1	(1.000,1.000)	1	(1.000,1.000)
No partner							1.019	(0.928,1.119)	0.992	(0.822,1.197)
**Number of living children**							0.973	(0.945,1.002)	0.978	(0.940,1.018)
**Self-perceived health**										
Poor							1	(1.000,1.000)	1	(1.000,1.000)
Good							1.336***	(1.238,1.442)	1.122	(0.992,1.269)
**Household size**							0.914***	(0.870,0.961)	0.916***	(0.872,0.963)
**Sex x Marital status**										
Women x with partner									1	(1.000,1.000)
Men x no partner									0.741**	(0.619,0.888)

	Model 1: Unconditional mean model (n=34838) Odds Ratio (95% Confidence Interval)		Model 2: Unconditional growth model (n=34838) Odds Ratio (95% Confidence Interval)		Model 2A: + time-constant variables (n=34838) Odds Ratio (95% Confidence Interval)		Model 2B: + time variant variables (n=34838) Odds Ratio (95% Confidence Interval)		Model 3 (n=34838) Odds Ratio (95% Confidence Interval)	
**Region x Sex**										
Central x women									1	(1.000,1.000)
South x men									0.692***	(0.561,0.853)
North x men									1.228*	(1.017,1.484)
**Region x Education**										
Central x low									1	(1.000,1.000)
South x middle									1.640***	(1.260,2.136)
South x high									1.269	(0.897,1.795)
North x middle									1.106	(0.885,1.382)
North x high									0.95	(0.752,1.199)
**Region x Marital status**										
Central x with partner									1	(1.000,1.000)
South x no partner									0.631*	(0.437,0.911)
North x no partner									1.547**	(1.144,2.091)
**Region x Number of children**										
South x number of children									0.917*	(0.853,0.987)
Central x number of children									1	(1.000,1.000)
North x number of children									1.041	(0.973,1.114)
**Part ii: For time slope**										
**Time**			0.926***	(0.917,0.934)	0.964***	(0.944,0.984)	0.946***	(0.927,0.967)	0.916***	(0.893,0.940)
**Age x Time**					0.997***	(0.996,0.998)	0.998***	(0.997,0.999)		
**Sex x Time**										
Women x time					1	(1.000,1.000)	1	(1.000,1.000)		
Men x time					0.993	(0.978,1.008)	0.993	(0.978,1.008)		
**Sex x Age x Time**										
Women x age x time									0.998**	(0.997,1.000)
Men x age x time									0.998***	(0.997,0.999)
**Region x Time**										
South x time					0.973*	(0.953,0.994)	0.975*	(0.955,0.996)	0.975*	(0.952,0.998)
Central x time					1	(1.000,1.000)	1	(1.000,1.000)	1	(1.000,1.000)
North x time					0.979*	(0.961,0.996)	0.979*	(0.962,0.996)	0.991	(0.970,1.012)
**Education x Time**										
Low x time					1	(1.000,1.000)	1	(1.000,1.000)	1	(1.000,1.000)
Middle x time					1.012	(0.994,1.031)	1.011	(0.993,1.030)	1.011	(0.993,1.030)
High x time					1.026*	(1.005,1.046)	1.023*	(1.002,1.044)	1.021*	(1.001,1.042)
**Self-perceived health x Time**										
Poor x time									1	(1.000,1.000)

	Model 1: Unconditional mean model (n=34838) Odds Ratio (95% Confidence Interval)		Model 2: Unconditional growth model (n=34838) Odds Ratio (95% Confidence Interval)		Model 2A: + time-constant variables (n=34838) Odds Ratio (95% Confidence Interval)		Model 2B: + time variant variables (n=34838) Odds Ratio (95% Confidence Interval)		Model 3 (n=34838) Odds Ratio (95% Confidence Interval)	
Good x time									1.030***	(1.013,1.048)
**Marital status x Time**										
With partner x time									1	(1.000,1.000)
No partner x time									1.026*	(1.003,1.050)
**Marital status x Region x Time**										
With partner x central x time									1	(1.000,1.000)
No partner x south x time									1.035	(0.987,1.085)
No partner x north x time									0.952*	(0.916,0.990)
Random Effect										
Intercept	1.719	(1.592,1.857)	2.873	(2.517,3.279)	2.352	(2.039,2.713)	2.319	(2.009-2.678)	2.26	(1.954,2.613)
Time slope			0.023	(0.019,0.028)	0.022	(0.018,0.027)	0.022	(0.018,0.027)	0.021	(0.017,0.026)
Covariance			-0.119	(-0.154,-0.083)	-0.136	(-0.170,-0.102)	-0.137	(-0.170,-0.103)	-0132	(-0.166,-0.099)
Intraclass correlation	0.343		0.466		0.417		0.414		0.407	

B. Type of instrumental support and recipients of instrumental support

Table B1. Type of instrumental support provided by wave and region

Type of Instrumental support provided	Wave	South		Central		North	
		n	%	n	%	n	%
Personal care	1	231	45.38	287	17.45	139	13.52
**Practical household help**	**1**	**336**	**66.01**	**1315**	**79.94**	**899**	**87.45**
Paperwork	1	187	36.74	599	36.41	345	33.56
Personal care	2	194	42.45	309	20.22	151	17.22
**Practical household help**	**2**	**305**	**66.74**	**1262**	**82.59**	**750**	**85.52**
Paperwork	2	170	37.2	525	34.36	282	32.16
Personal care	6	95	40.95	238	18.49	90	12.41
**Practical household help**	**6**	**142**	**61.21**	**1077**	**83.68**	**634**	**87.45**
Paperwork	6	93	40.09	395	30.69	167	23.03

Note: Data on type of support was only available in wave 1,2 and 6.

Table B2. Type of relationship with support recipient by wave and region

Type of Instrumental support provided	Wave	South		Central		North	
		n	%	n	%	n	%
**Parents/children/partner**	**1**	**278**	**54.62**	**1055**	**64.02**	**720**	**70.04**
Friends & neighbour	1	142	27.9	457	27.73	351	34.14
Extended family /other relationships	1	163	32.02	405	24.58	209	20.33
**Parents/children/partner**	**2**	**255**	**55.68**	**985**	**64.17**	**629**	**71.72**
Friends & neighbour	2	126	27.51	419	27.3	251	28.62
Extended family /other relationships	2	131	28.6	357	23.26	166	18.93
**Parents/children/partner**	**4**	**118**	**52.21**	**484**	**57.08**	**313**	**61.49**
Friends & neighbour	4	60	26.55	326	38.44	201	39.49
Extended family /other relationships	4	82	36.28	193	22.76	103	20.24
**Parents/children/partner**	**5**	**106**	**45.89**	**455**	**53.72**	**342**	**61.96**
Friends & neighbour	5	68	29.44	346	40.85	236	42.75
Extended family /other relationships	5	87	37.66	223	26.33	106	19.20
**Parents/children/partner**	**6**	**121**	**51.93**	**687**	**53.3**	**413**	**56.97**
Friends & neighbour	6	64	27.47	511	39.64	324	44.69
Extended family /other relationships	6	66	28.33	288	22.34	106	14.62

C. Interaction Plots

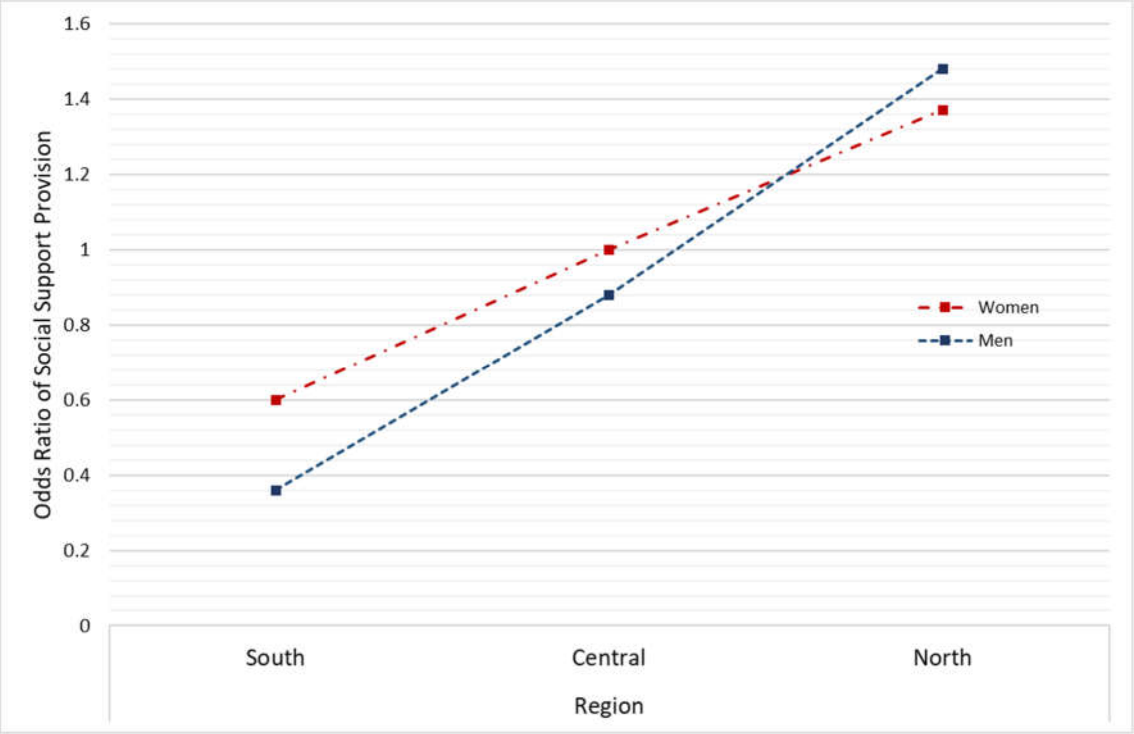

Fig. C1. Interaction plot of sex and region on the odds of instrumental support provision

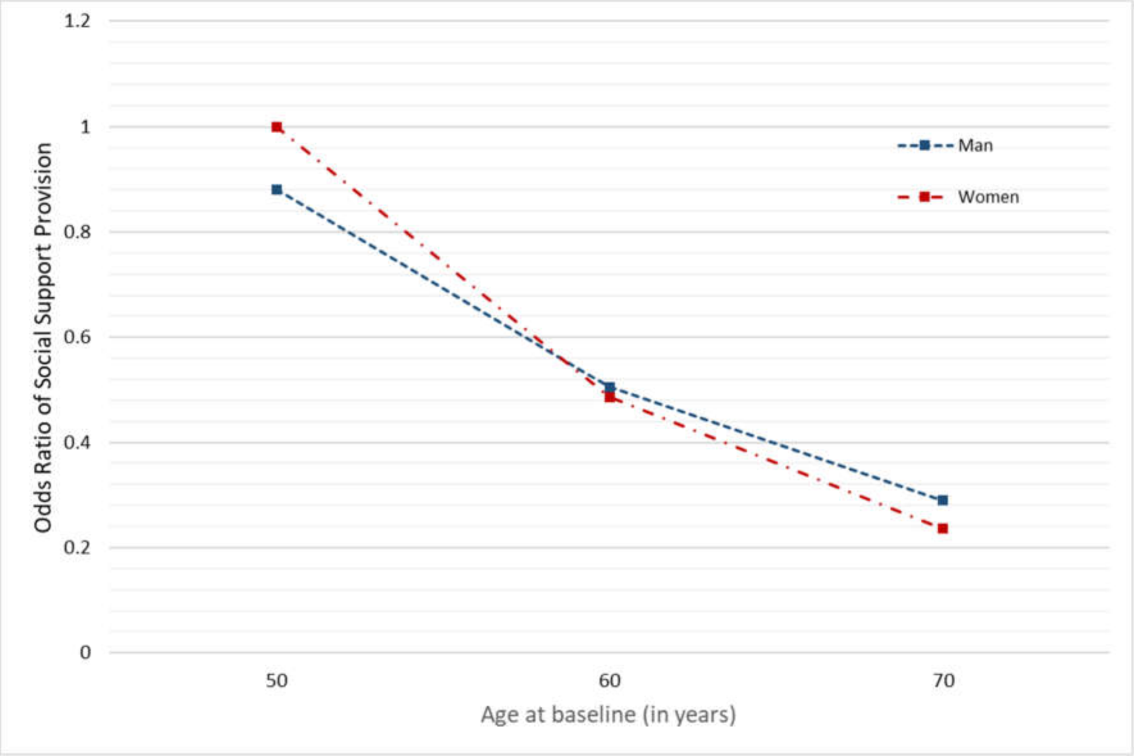

Fig. C2. Interaction plot of sex and age at baseline on the odds of instrumental support provision

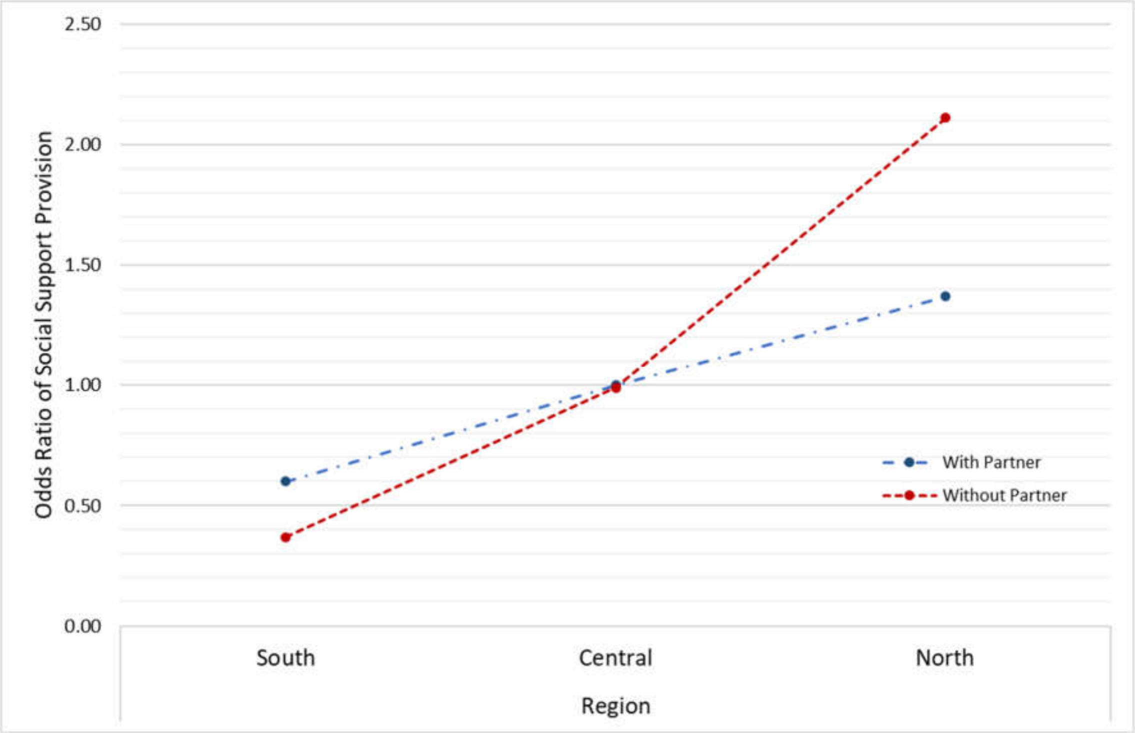

Fig. C3. Interaction plot of marital status and region on the odds of instrumental support provision

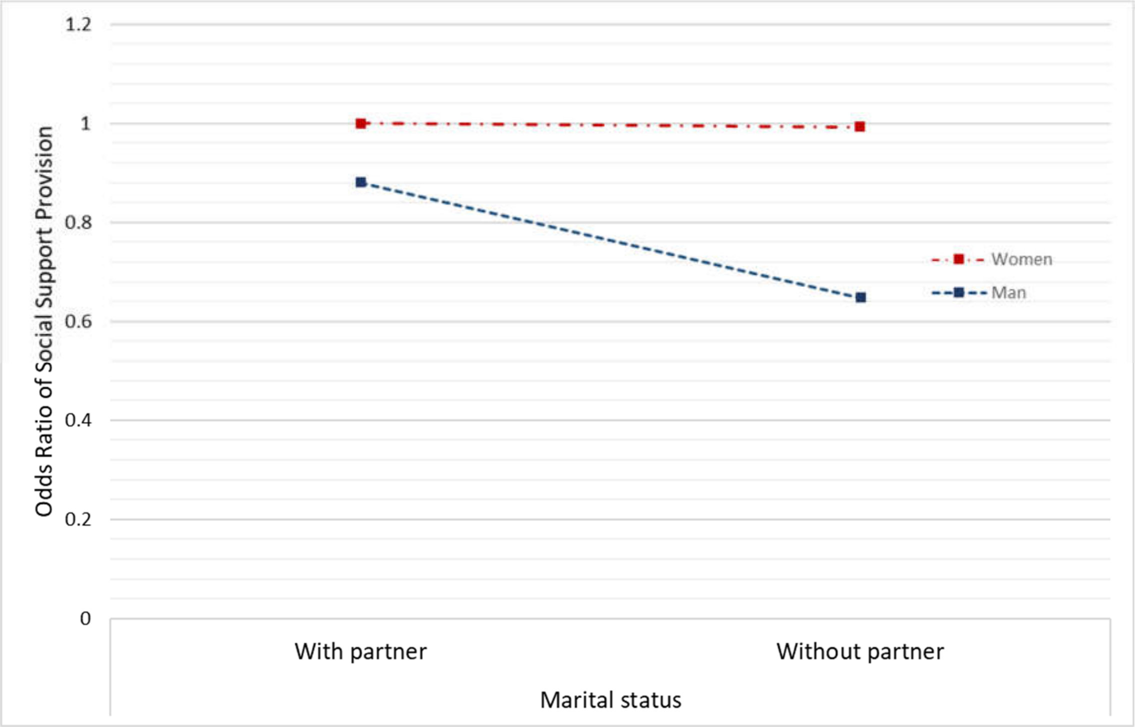

Fig. C4. Interaction plot of sex and marital status on the odds of instrumental support provision

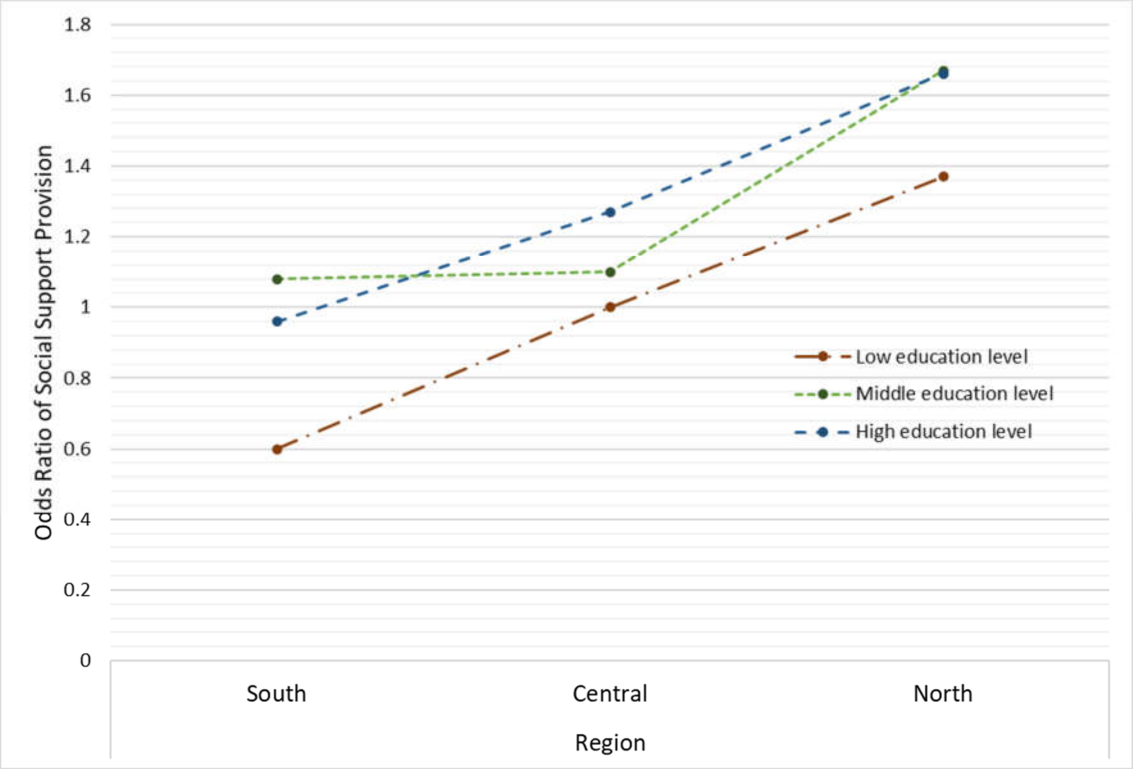

Fig. C5. Interaction plot of education level and region on the odds of instrumental support provision

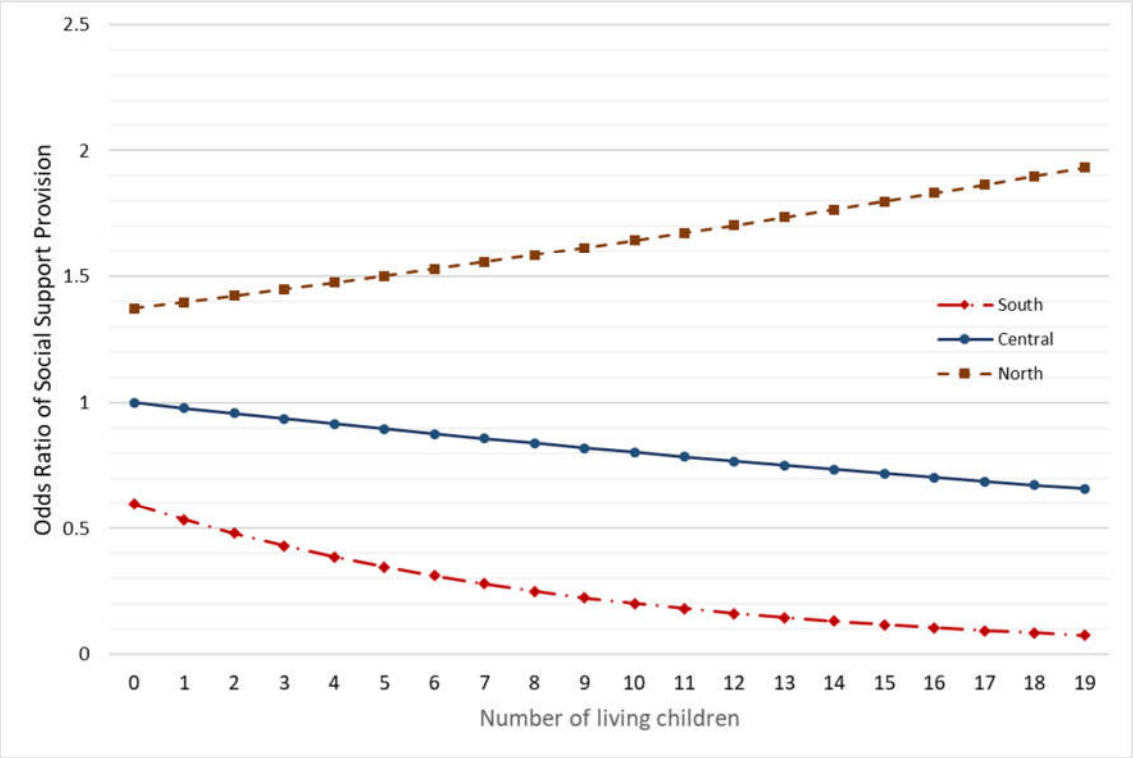

Fig. C6. Interaction plot of number of living children and region on the odds of instrumental support provision

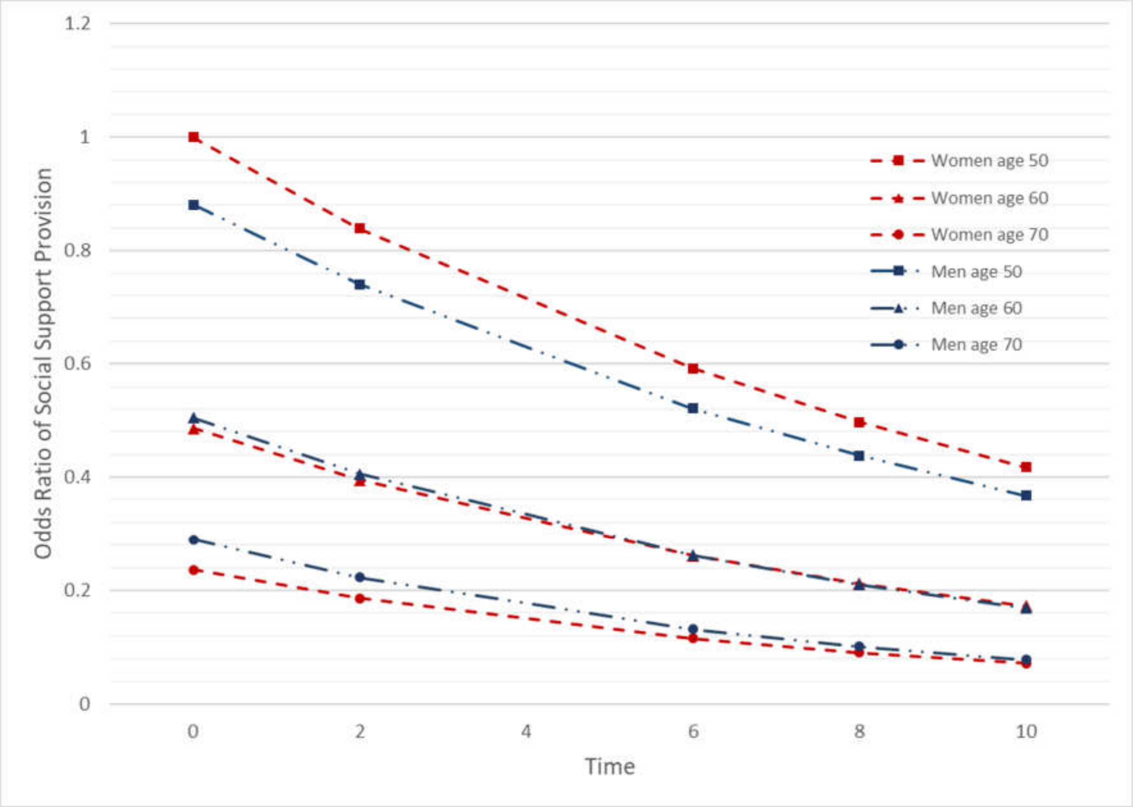

Fig. C7. Interaction plot of sex, age at baseline and time on the odds of instrumental support provision

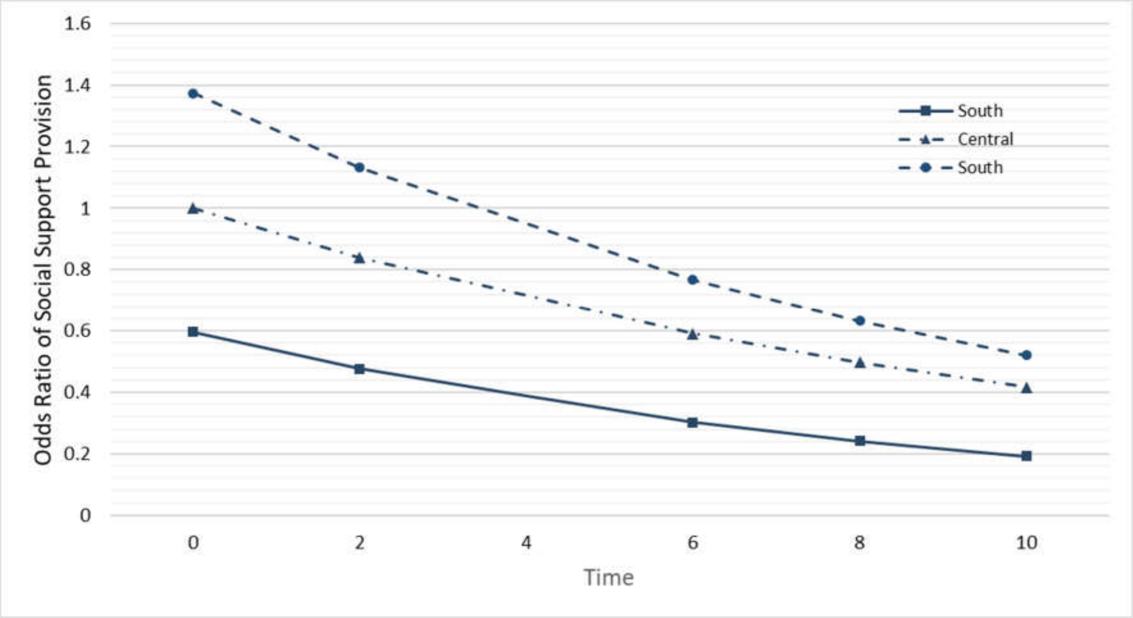

Fig. C8. Interaction plot of region and time on the odds of instrumental support provision

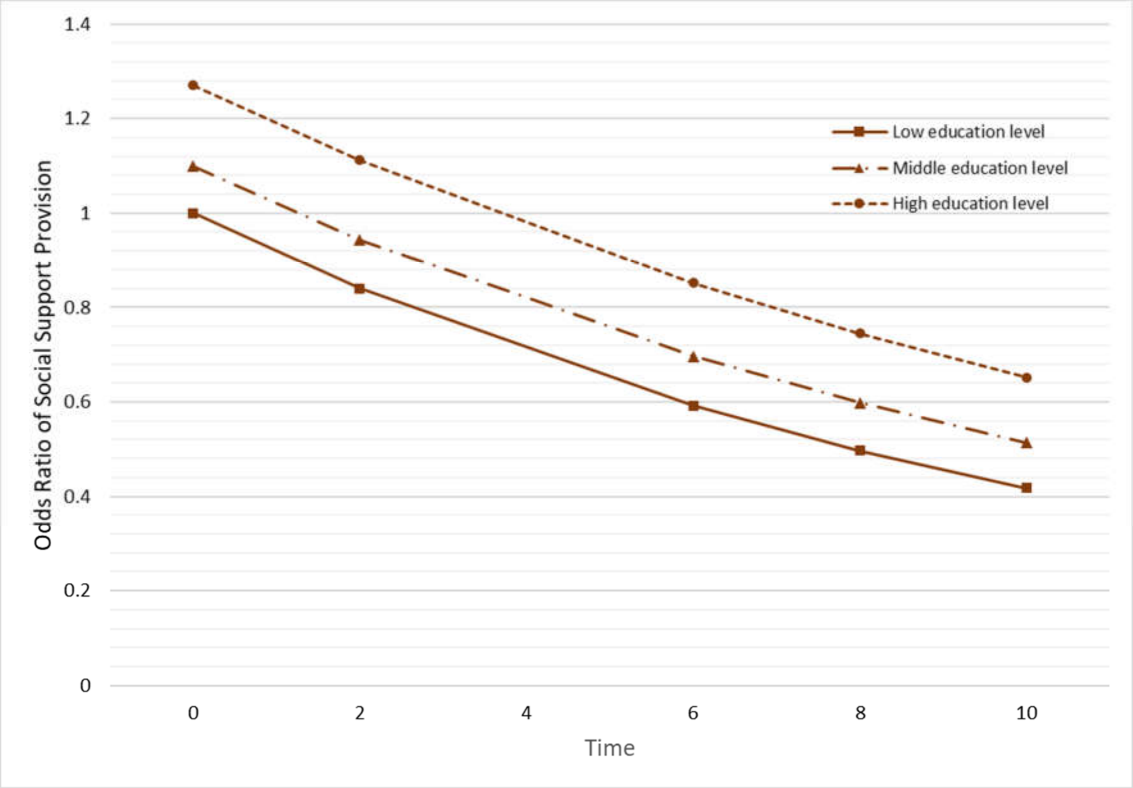

Fig. C9. Interaction plot of highest education level and time on the odds of instrumental support provision

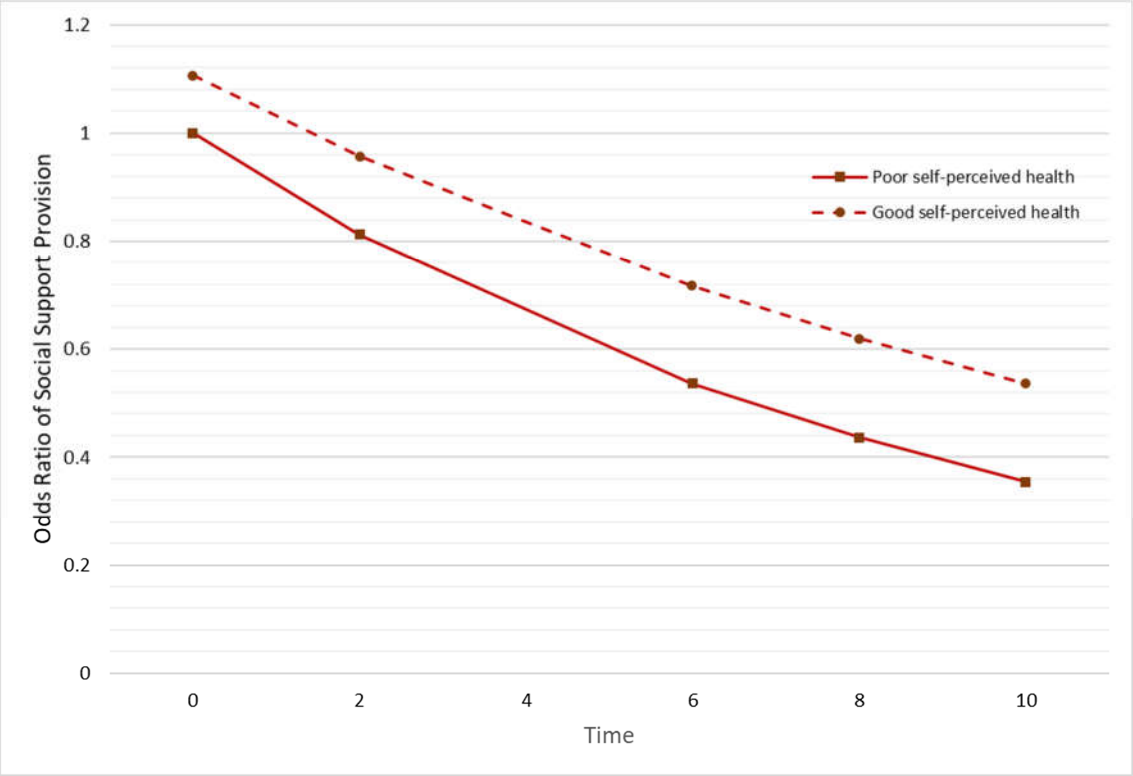

Fig. C10. Interaction plot of self-perceived health and time on the odds of instrumental support provision

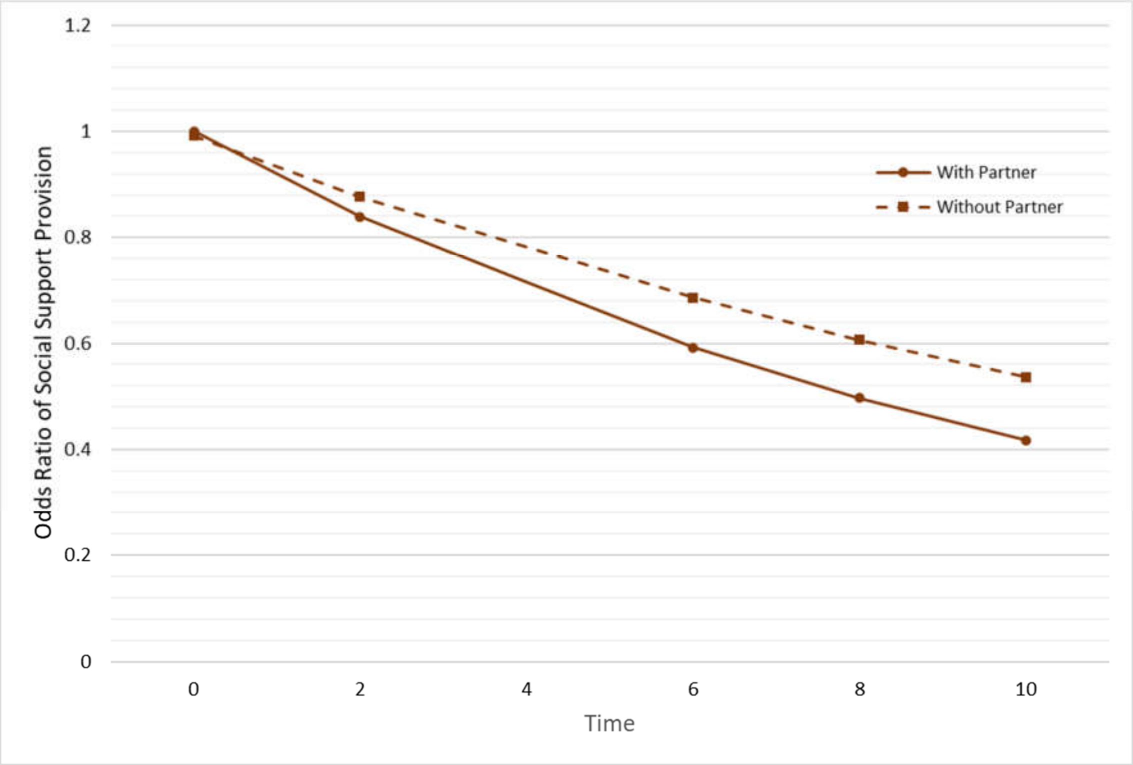

Fig. C11. Interaction plot of marital status and time on the odds of instrumental support provision

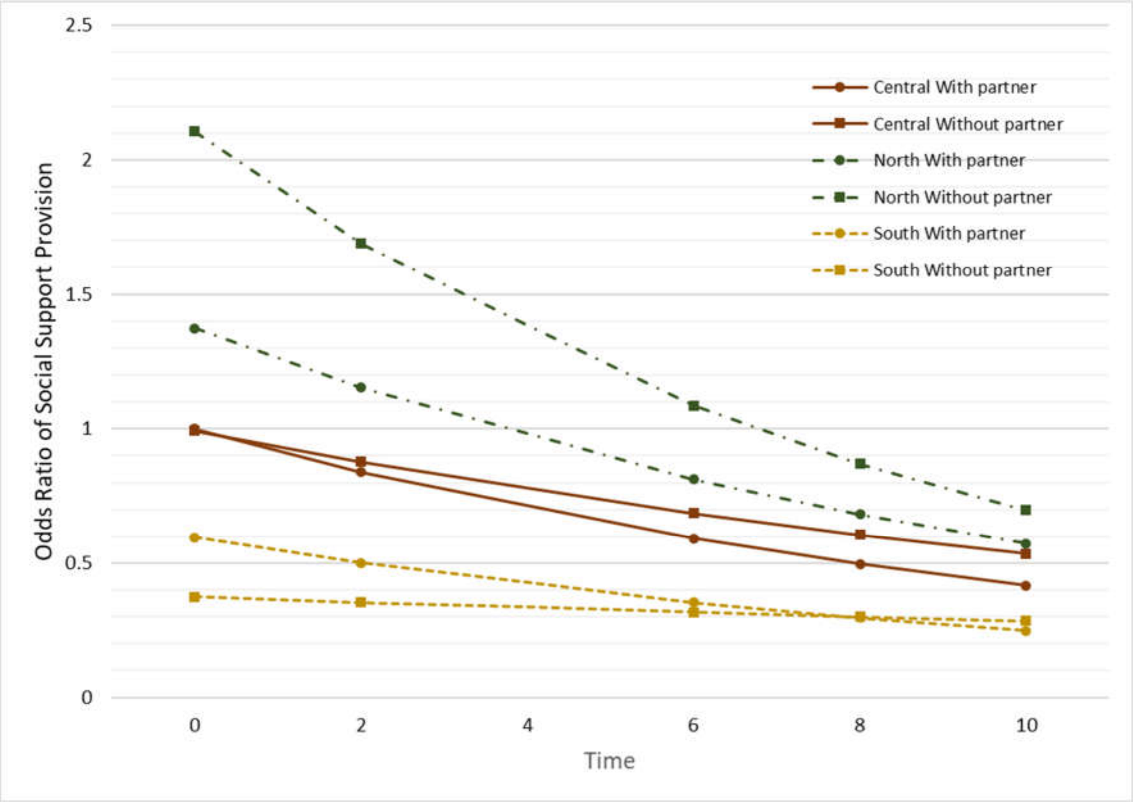

Fig. C12. Interaction plot of region, marital status, and time on the odds of instrumental support provision

D. Predicted probability plots

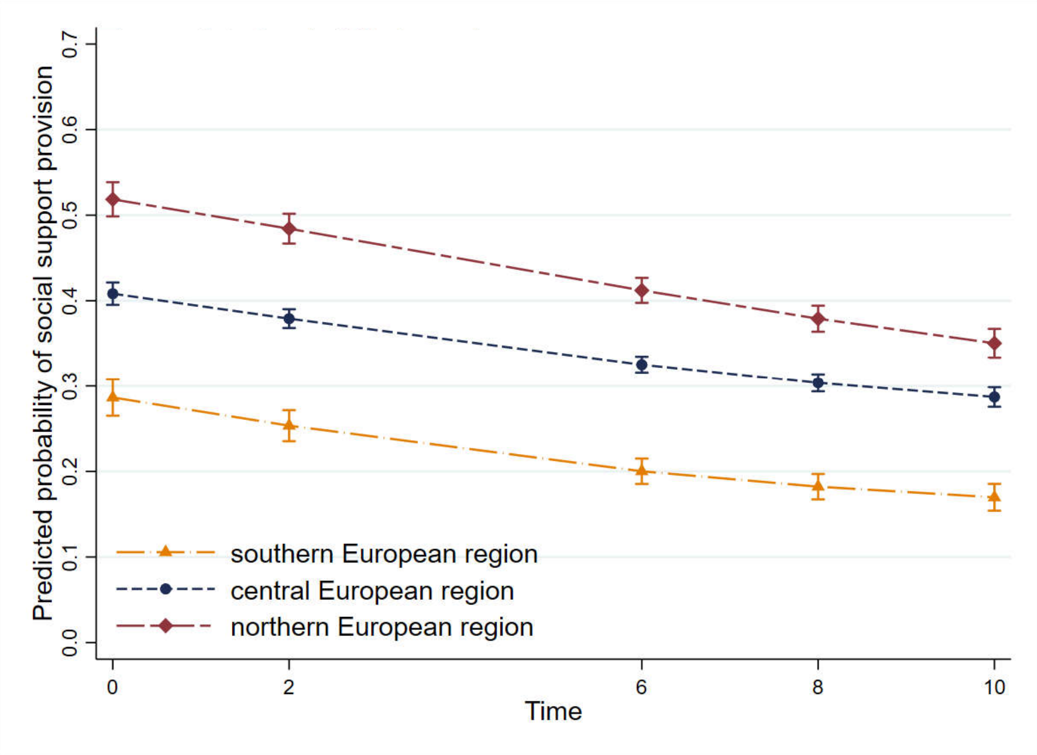

Fig. D1. Predicted probability of instrumental support provision by region

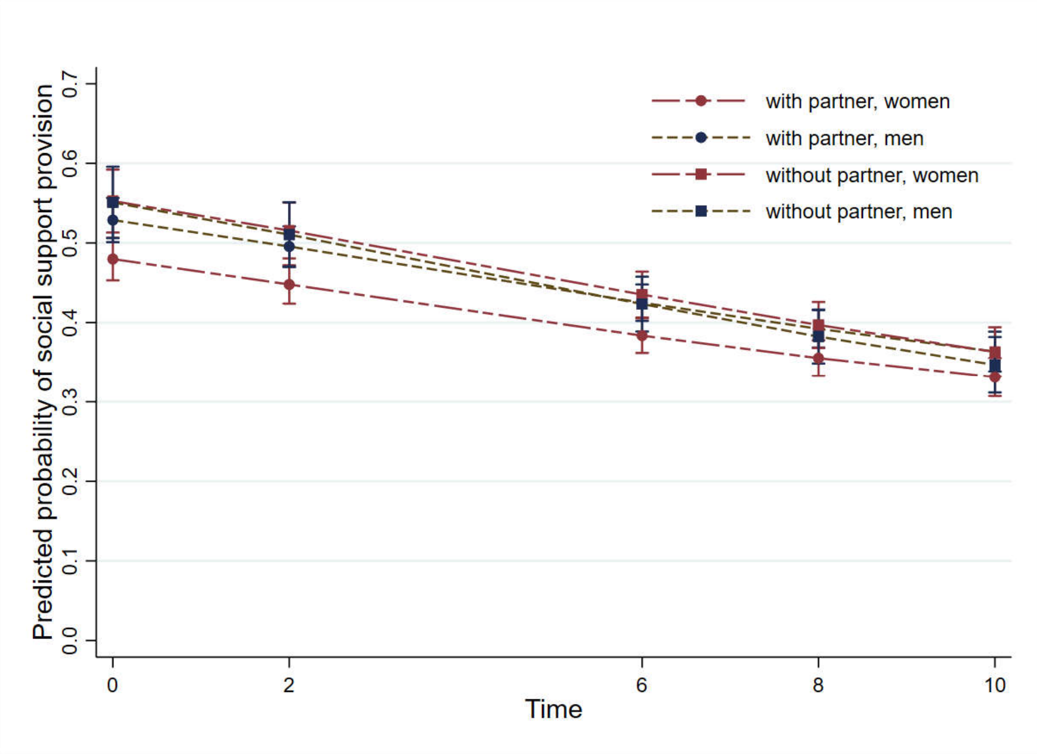

Fig. D2. Predicted probability of instrumental support provision by marital status in Northern Europe

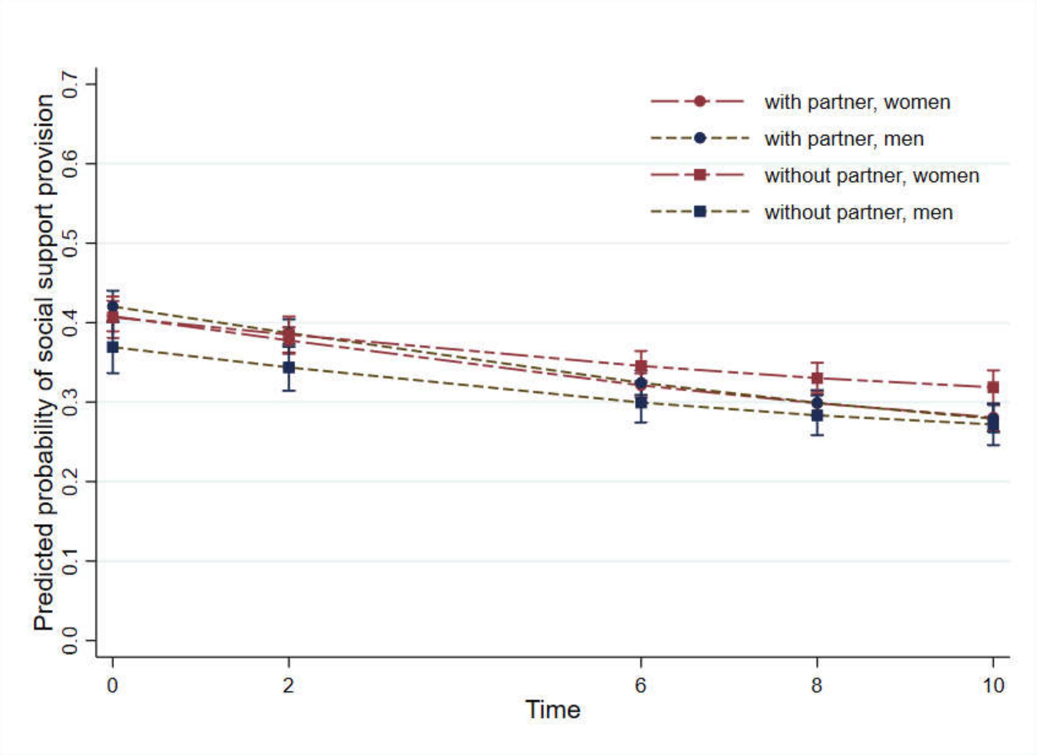

Fig. D3. Predicted probability of instrumental support provision by marital status in Central Europe

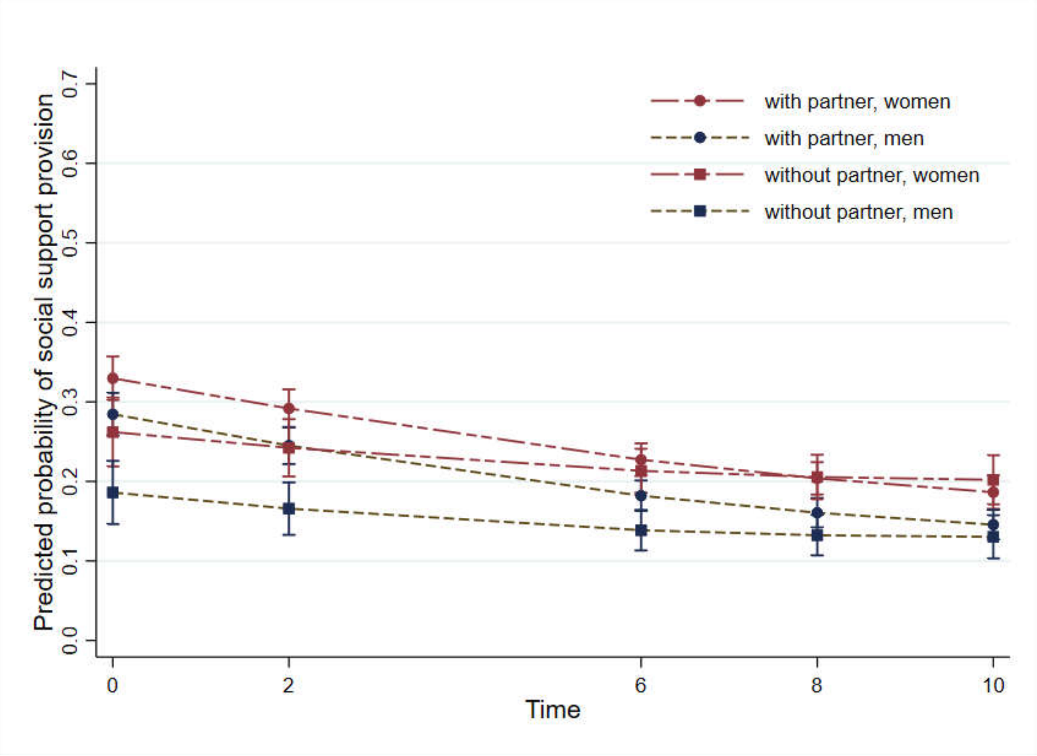

Fig. D4. Predicted probability of instrumental support provision by marital status in Southern Europe

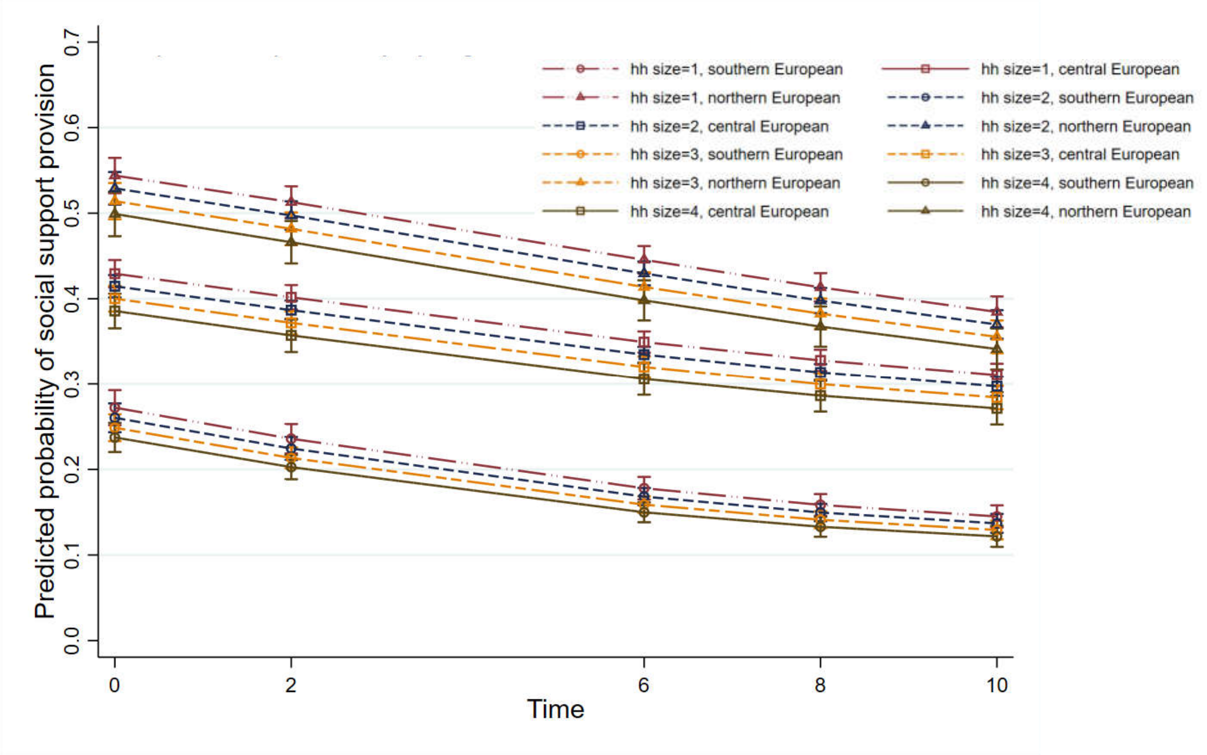

Fig. D5. Predicted probability of instrumental support provision by household size over region

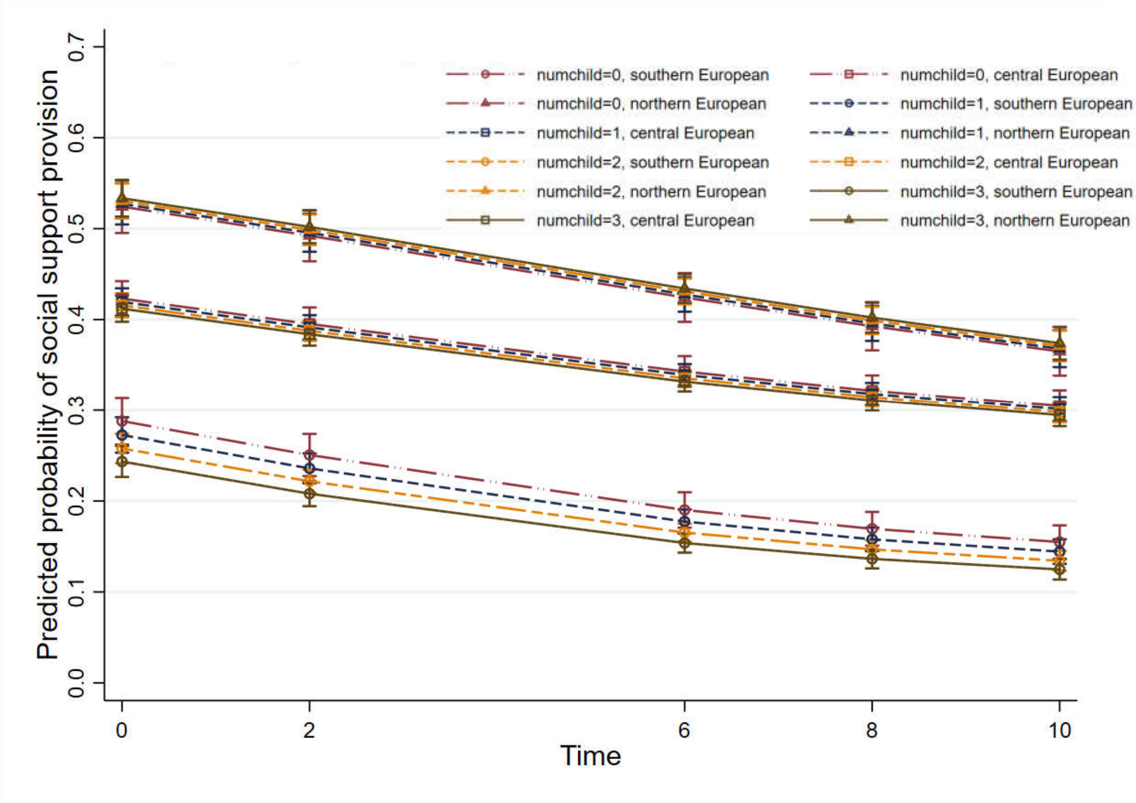

Fig. D6. Predicted probability of instrumental support provision by number of living children over region

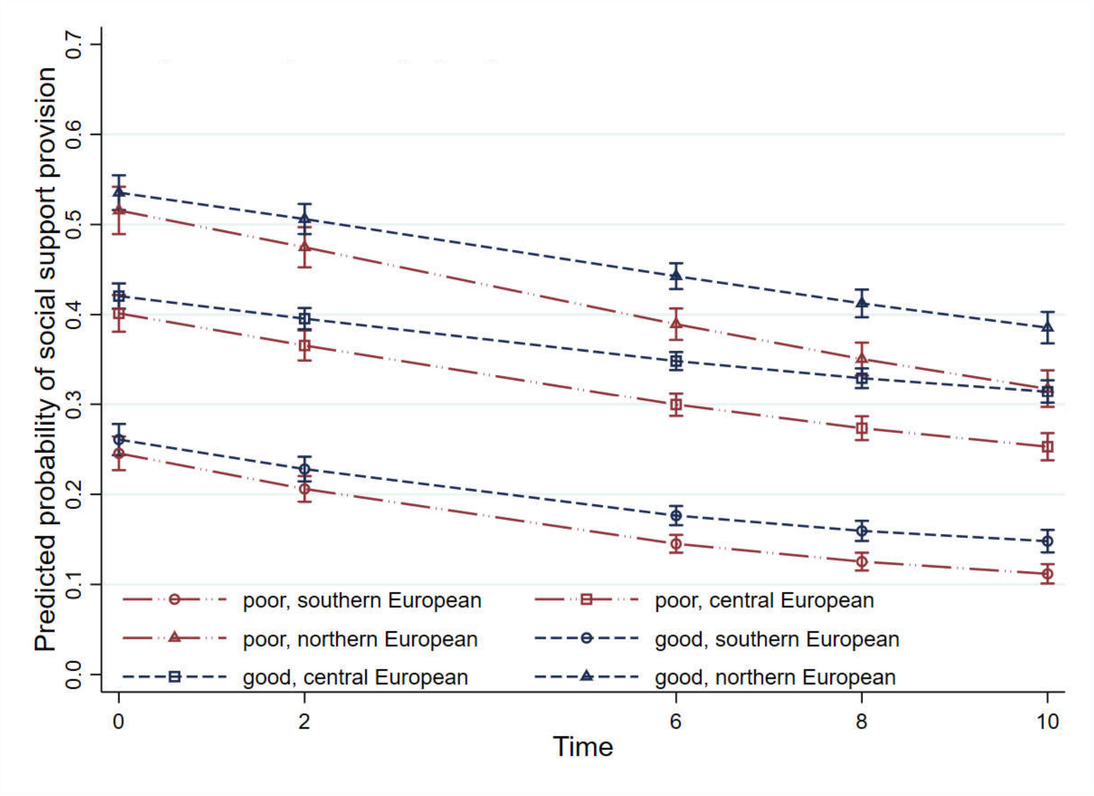

Fig. D7. Predicted probability of instrumental support provision by self-perceived health over region

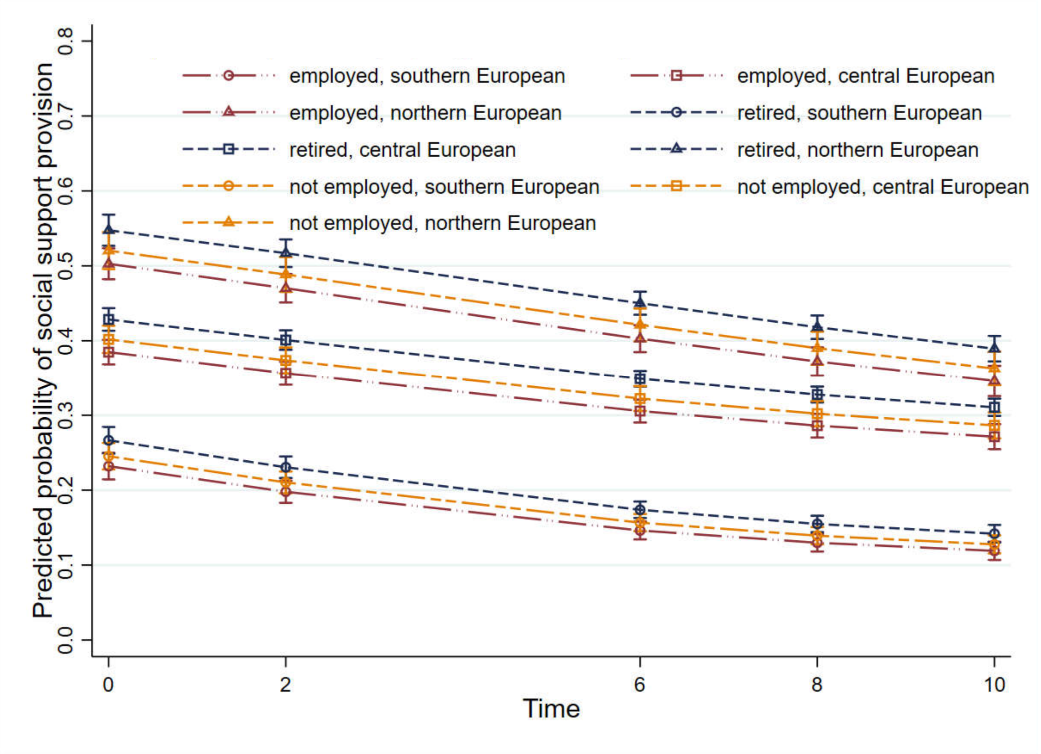

Fig. D8. Predicted probability of instrumental support provision by employment status over region

E. Characteristics of sample and non-sample

Table E1. Characteristics of the eligible sample* and the final sample

	Eligible sample	Final sample	Total	p-value
**Age at baseline, (Mean ±s.d), n=8636)**	61.6 ± 8.1	62.5 ± 8.2	62.4 ± 8.2	0.1012 [a]
**Number living children, (Mean ±s.d), n=8620)**	2.4 ± 1.6	2.2 ± 1.4	2.3 ± 1.4	0.0458 [a]
**Household size, (Mean ±s.d), n=8636)**	2.5 ± 1.0	2.3 ± 1,0	2.3 ± 1.0	0.0003 [a]
**Region (n(%), n=8636)**				
South	77 (27.3)	2213 (26.5)	2290 (26.52)	0.794 [b]
Central	134 (47.5)	4139 (49.6)	4273 (49.48)	
North	71 (25.2)	2002 (24.0)	2073 (24)	
**Sex (n(%), n=8636)**				
Women	144 (51.0)	4704 (56.3)	4848 (56.14)	0.081 [b]
Men	138 (48.9)	3650 (43.7)	3788 (43.86)	
**Education (n(%), n=8571)**				
Low	119 (54.8)	4010 (48.0)	4129 (48.17)	0.134 [b]
Middle	58 (26.7)	2515 (30.1)	2573 (30.02)	
High	40 (18.4)	1829 (21.9)	1869 (21.81)	
**Marital Status (n(%), n=8623)**				
With partner	238 (87.5)	6360 (76.2)	6598 (76.52)	0.000 [b]
Without partner	34 (12.5)	1991 (23.9)	2025 (23.48)	
**Employment Status (n(%), n=8600)**				
Employed	109 (41.9)	2813 (33.7)	2922 (33.98)	0.023 [b]
Retired	101 (38.9)	3715 (44.5)	3816 (44.37)	
Not employed	50 (19.2)	1812 (21.7)	1862 (21.65)	
**Self-perceived health (n(%), n=8612)**				
Poor	49 (18.3)	1919 (23.0)	1968 (22.85)	0.070 [b]
Good	219 (81.7)	6425 (77.0)	6644 (77.15)	
**Provided social support (n(%), n=8593)**				
Yes	87 (33.5)	3186 (38.2)	3273 (38.09)	0.119 [b]
No	173 (66.5)	5147 (61.8)	5320 (61.91)	

p-value was obtained from [a] two-sample t-test and [b] Chi-squared test
* aged 50 and over in wave 1; participated in at least waves 1 and 6; and have never moved to a nursing home.

Table E2. Characteristics of SHARE sample (age ≥ 50 from the nine countries) and the final sample

	SHARE sample	Final sample	Total	p-value
**Age at baseline, (Mean ±s.d), n=21483)**	66.9 ± 10.8	62.5 ± 8.2	65.2 ± 10.1	0.000 [a]
**Number living children, (Mean ±s.d), n=21423)**	2.2 ± 1.6	2.2 ± 1.4	2.2 ± 1.5	0.000 [a]
**Household size, (Mean ±s.d), n=21483)**	2.1 ± 1.0	2.3 ± 1,0	2.2 ± 1.0	0.000 [a]
**Region (n(%), n=21483)**				
South	2573 (19.6)	2213 (26.5)	4786 (22.3)	0.000 [b]
Central	7943 (60.5)	4139 (49.6)	12082 (56.2)	
North	2613 (19.9)	2002 (24.0)	4615 (21.5)	
**Sex (n(%), n=21483)**				
Women	7016 (53.4)	4704 (56.3)	11720 (54.6)	0.000 [b]
Men	6113 (46.6)	3650 (43.7)	9763 (45.5)	
**Education (n(%), n=21283)**				
Low	6771 (52.4)	4010 (48.0)	10781 (50.7)	0.000 [b]
Middle	4026 (31.1)	2515 (30.1)	6541 (30.7)	
High	2132 (16.5)	1829 (21.9)	3961 (18.6)	
**Marital Status (n(%), n=21431)**				
With partner	9123 (69.8)	6360 (76.2)	15483 (72.25)	0.000 [b]
Without partner	3957 (30.3)	1991 (23.9)	5948 (27.75)	
**Employment Status (n(%), n=21431)**				
Employed	3020 (23.2)	2813 (33.7)	5833 (27.3)	0.000 [b]
Retired	7307 (56.2)	3715 (44.5)	11022 (51.7)	
Not employed	2674 (20.6)	1812 (21.7)	4486 (21.0)	
**Self-perceived health (n(%), n=21378)**				
Poor	4557 (35.0)	1919 (23.0)	6476 (30.3)	0.000 [b]
Good	8477 (65.0)	6425 (77.0)	14902 (69.7)	
**Provided social support (n(%), n=21315)**				
Yes	3749 (28.9)	3186 (38.2)	6935 (32.5)	0.000 [b]
No	9233 (71.1)	5147 (61.8)	14380 (67.5)	

p-value was obtained from [a] two-sample t test and [b] Chi-squared test
